# Dissecting the Solid Polymer Electrolyte–Electrode Interface in the Vicinity of Electrochemical Stability Limits

**DOI:** 10.1021/acsami.2c02118

**Published:** 2022-06-16

**Authors:** Christofer Sångeland, Guiomar Hernández, Daniel Brandell, Reza Younesi, Maria Hahlin, Jonas Mindemark

**Affiliations:** ‡ Department of Chemistry—Ångström Laboratory, Uppsala University, Box 538, SE-751 21 Uppsala, Sweden; † Department of Physics and Astronomy, Uppsala University, Box 516, SE-751 20 Uppsala, Sweden

**Keywords:** lithium-ion batteries, solid polymer electrolytes, electrochemical stability window, solid electrolyte interphase, cathode electrolyte interphase, electrochemical impedance spectroscopy, X-ray photoelectron spectroscopy

## Abstract

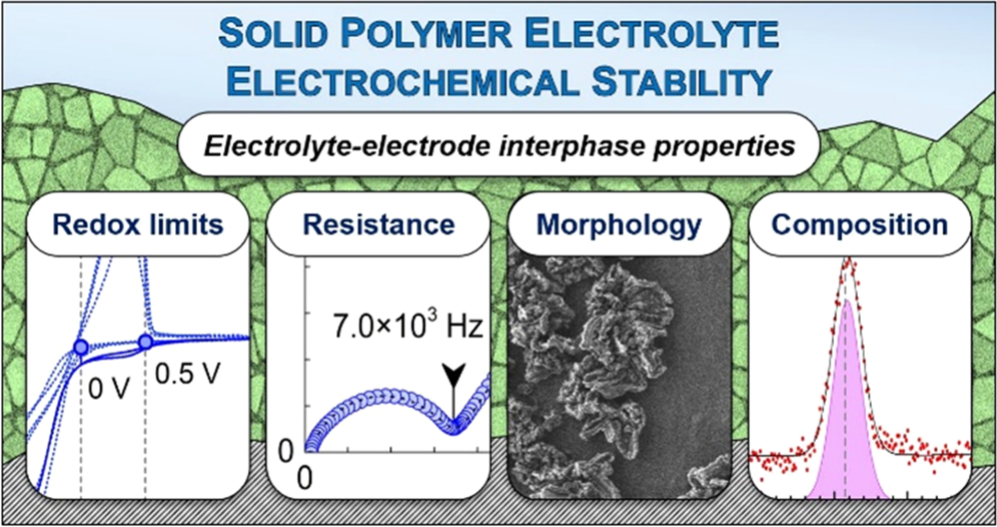

Proper understanding of solid polymer electrolyte–electrode interfacial layer formation and its implications on cell performance is a vital step toward realizing practical solid-state lithium-ion batteries. At the same time, probing these solid–solid interfaces is extremely challenging as they are buried within the electrochemical system, thereby efficiently evading exposure to surface-sensitive spectroscopic methods. Still, the probing of interfacial degradation layers is essential to render an accurate picture of the behavior of these materials in the vicinity of their electrochemical stability limits and to complement the incomplete picture gained from electrochemical assessments. In this work, we address this issue in conjunction with presenting a thorough evaluation of the electrochemical stability window of the solid polymer electrolyte poly(ε-caprolactone):lithium bis(trifluoromethanesulfonyl)imide (PCL:LiTFSI). According to staircase voltammetry, the electrochemical stability window of the polyester-based electrolyte was found to span from 1.5 to 4 V vs Li^+^/Li. Subsequent decomposition of PCL:LiTFSI outside of the stability window led to a buildup of carbonaceous, lithium oxide and salt-derived species at the electrode–electrolyte interface, identified using postmortem spectroscopic analysis. These species formed highly resistive interphase layers, acting as major bottlenecks in the SPE system. Resistance and thickness values of these layers at different potentials were then estimated based on the impedance response between a lithium iron phosphate reference electrode and carbon-coated working electrodes. Importantly, it is only through the combination of electrochemistry and photoelectron spectroscopy that the full extent of the electrochemical performance at the limits of electrochemical stability can be reliably and accurately determined.

## Introduction

The implementation of lithium metal anodes and high-voltage cathodes promises the inception of next-generation lithium-ion batteries (LIBs) with gravimetric energy densities exceeding 260 Wh kg^–1^.^1^ However, state-of-the-art liquid electrolytes are incompatible with these electrodes, mainly due to their inability to prevent lithium dendrite growth and electrode cross-talk,^1−3^ therefore necessitating a new class of electrolyte materials. In this regard, nonvolatile solid polymer electrolytes (SPEs) are promising alternatives due to their wettability (interfacial contact) and scalability.^4,5^ Moreover, SPEs grant us the opportunity to tailor the properties of the electrolyte locally. For example, the electrochemical stability window (ESW) of the electrolyte can be significantly extended by assembling a double-layer SPE, composed of one layer, which is stable toward the anode and one which is stable toward the cathode.^6^ Previously, the operating temperature of solid polymer electrolyte LIBs has been restricted to elevated temperatures due to the sub-par ionic conductivity of polyether-based electrolytes.^7^ Nonetheless, recent diversification of available polymer hosts has overcome this hurdle.^7^ For example, poly(ε-caprolactone-*co*-trimethylene carbonate) (PCL–PTMC) exhibits a low glass-transition temperature, low degree of crystallinity, and a weaker affinity for lithium ions, properties that favor lithium-ion transport.^8,9^ Accordingly, a respectable ionic conductivity of 4.1 × 10^–5^ S cm^–1^ at 25 °C and transference number of 0.62 at 40 °C was achieved using this material when combined with LiTFSI.^10^ The practical application of this SPE has further been demonstrated in a LiFePO_4_ (LFP) half-cell capable of operating at ambient temperature.^10^ Likewise, PCL can also be blended together with PEO to obtain an electrolyte host with tunable properties dependent on the ratio of polymer constituents.^11^

In liquid electrolyte LIBs, the formation of kinetically stable and highly ion-conductive degradation layers, e.g., the solid electrolyte interphase (SEI), is critical to ensure high capacity retention and high-power capability.^12^ Likewise, resistances associated with interphase formation may in some cases be major bottlenecks in all-solid-state LIBs.^13−16^ Despite this, little research has been devoted to understanding the passivation and degradation mechanisms that occur at the solid electrolyte–electrode interface of these systems.^15^ Typically, the ESW of SPEs is defined based on the current response observed using voltammetry techniques.^17^ However, there exists no widely adopted definition for how to reliably determine the oxidation and reduction onsets.^17,18^ Furthermore, the stability of SPEs is tested against a wide range of different working electrodes (WE), scan rates, and temperatures.^17,18^ This is problematic since area and catalytic effects of the working electrode surface will influence the current response.^19^ Concerning PCL–PTMC:LiTFSI, the electrochemical stability and interphase species of the polycarbonate component have been characterized using linear sweep voltammetry (LSV), X-ray photoelectron spectroscopy (XPS), and computational simulations, but we have yet to investigate the influence of the polyester subunits.^20−23^ From a practical perspective, obtaining reliable samples of this material for postmortem surface analysis is challenging due to its adhesive quality. For this reason, we may adjust the focus slightly to solely investigate the stability of PCL:LiTFSI, which is similar to PCL–PTMC:LiTFSI, but less adhesive. Previously, the ESW of PCL:LiTFSI was estimated to range from 0.3 to 5.4 V vs Li^+^/Li using a stainless steel working electrode at 60 °C.^24^ Atomistic modeling estimated a similar anodic limit; however, the cathodic limit was estimated to be 1.2 V vs Li^+^/Li.^22^ Using molecular dynamics, Ebadi et al. showed that the C_carbonyl_–O_ester_ bond in PCL is prone to breakage in the presence of lithium metal.^23^ Eriksson et al. demonstrated that by adding Al_2_O_3_ nanoparticles to PCL:LiTFSI, it was possible to stabilize the SPE–lithium metal interface under static conditions at 30 °C.^25^ In this work, we have synthesized a high-molecular-weight PCL to use as a less adhesive mimic with similar structural and electrochemical properties to the high-performance PCL–PTMC material, sharing a largely identical polymer backbone. Combining this with a lower salt concentration and testing temperature, a material is obtained which facilitates separation from the electrode for postmortem compositional and morphological analysis. The electrochemical stability of PCL:LiTFSI was evaluated using a collection of complementing techniques to dissect the behavior of the SPE–electrode interface in the vicinity of the stability limits. Our aim with this study is to provide context to the reduction and oxidation currents and what they entail for the SPE–electrode interface in terms of resistance, morphology, and composition.

## Experimental Section

### Materials

ε-Caprolactone monomer (CL; Perstorp) was dried by distillation over CaH_2_ under reduced pressure. Lithium bis(trifluoromethanesulfonyl)imide salt (LiTFSI; BASF) was dried at 120 °C under vacuum for 48 h. Tin(II)-2-ethylhexanoate (SnOct_2_; Sigma), toluene (Acros Organics, Super dry with molecular sieves), tetrahydrofuran (THF; Sigma-Aldrich, anhydrous, inhibitor-free), carbon-coated copper foil (Cu–C; MTI), carbon-coated aluminum foil (Al–C; Showa Denko SDX), LiFePO_4_ (LFP_P.tech_; Phostech), carboxymethyl cellulose (CMC; Sigma-Aldrich), C65 (Imerys Graphite and Carbon), lithium metal foil (Cyprus Foote Mineral Co, 125 μm), aluminum foil with double-sided LiFePO_4_ coating (LiFeSiZE, 3.85 mAh cm^–2^), poly(propylene) separator (Celgard 2500, 25 μm), LP40 (Gotian, 1 M LiPF_6_ EC/DEC 1:1 vol.), and dimethyl carbonate (DMC; Sigma-Aldrich) were all used as-received and kept in inert argon atmosphere unless stated otherwise.

### Poly(ε-caprolactone) Synthesis

High-molecular-weight poly(ε-caprolactone) (PCL) was prepared via bulk ring-opening polymerization, which has been described in detail previously.^20^ In summary, ε-caprolactone monomer and SnOct_2_ catalyst were combined in a predried stainless steel reactor and polymerized in an oven at 130 °C for 72 h. After polymerization, the opaque and milky white product was removed while warm to allow the material to be cut into smaller pieces for later use. The molecular structure of the polymer was confirmed using ^1^H NMR on a JEOL ECZ 400S spectrometer. Peak positions matched those previously reported for polycaprolactone^24^ (see Figure S1). The molecular weight and polydispersity index of the polymer were determined using gel permeation chromatography performed at PSS Polymer Standards Service GmbH in Mainz, Germany vs polystyrene standards. The *M*_n_, *M*_w_, and *Đ*_M_ of the polymer were 386,000, 741,000 g mol^–1^, and 1.96, respectively.

### Polymer Electrolyte Film Preparation

PCL was dissolved in anhydrous THF with 20 wt % LiTFSI salt. The ratio of polymer to solvent was 50 mg mL^–1^. The solution was stirred for 12 h at 50 °C and then cast in large poly(tetrafluoroethylene) (PTFE) molds. The solvent was removed using a previously described vacuum and heating procedure.^8^ In summary, the pressure was reduced to 200 mbar during the first 2 min, followed by a slow decrease to <2 mbar over the next 20 h at 30 °C. Next, the temperature was increased to 60 °C and held for an additional 40 h at vacuum. To obtain films with homogeneous thickness, PCL:LITFSI was placed in an MTI 6T hydraulic lamination hot press between two PTFE sheets and preheated at 90 °C for 30 min. Thereafter, 25 MPa was applied for 30 min at the same temperature, after which the heater was turned off and the temperature cooled to 40 °C while maintaining pressure.

### Thermogravimetric Analysis (TGA)

To ensure that the polymer electrolyte did not chemically deteriorate during the hot-pressing step, the thermal stability of PCL:LiTFSI before hot pressing was evaluated using thermogravimetric analysis (TGA). The percentage weight loss was measured from 25 to 400 °C at a ramp rate of 5 °C min^–1^ under N_2_ flow on a TA Instruments TGA Q500. In addition, the thermal stability at elevated temperatures for a prolonged time was determined by stepping the temperature 20 °C every 3 h from 40 to 400 °C. Samples were briefly exposed to ambient conditions during transfer.

### Differential Scanning Calorimetry

To facilitate electrolyte–electrode separation for postmortem surface analysis, voltammetry measurements were carried out below the melting point of PCL:LiTFSI to prevent the polymer electrolyte from sticking to the electrodes. The melting point (*T*_m_) and glass-transition temperature (*T*_g_) of PCL and PCL:LITFSI were determined using a TA Instruments DSC Q2000 differential scanning calorimeter. Polymer samples (∼7.5 mg) were hermetically sealed in aluminum pans and cooled to −80 °C at 5 °C min^–1^ followed by thermal equilibration. Next, the pans were heated to 80 °C at 10 °C min^–1^ again followed by thermal equilibration. The thermal sweep was repeated once more, and *T*_g_ and *T*_m_ were extracted from the second scan using TA Instruments Universal Analysis 2000 v. 4.5A.

### Ionic Conductivity

The total ionic conductivity of PCL:LiTFSI was determined using electrochemical impedance spectroscopy (EIS). The thickness of the films (12 mm in diameter) was determined using a Mitutoyo digital indicator micrometer. Next, the films were hermetically sealed between two stainless steel blocking electrodes in coin cells (Hohsen, 2025) along with a PTFE spacer ring and annealed at 50 °C for 1 h to ensure good contact between the blocking electrodes and the polymer electrolyte. The impedance was measured using a Schlumberger impedance/Gain-Phase analyzer SI 1260 from 10^7^ to 1 Hz with an amplitude of 10 mV at intervals between 25 and 90 °C. The bulk electrolyte resistance was determined by fitting a Debye equivalent circuit (see Table S1) to the Nyquist plot in ZView v. 3.2b.^8^ Using the measured thickness, area, and electrolyte resistance, the total ionic conductivity was calculated.

### Voltammetry

Linear sweep voltammetry (LSV) and cyclic voltammetry (CV) measurements were done with a scan rate of 0.1 mV s^–1^ at 40 °C using a Bio-Logic SP240 portable potentiostat. Cells consisting of a lithium metal counter electrode (CE) (17 mm in diameter), PCL:LiTFSI polymer electrolyte (22 mm in diameter), and a Cu–C or Al–C working electrode (17 in diameter) were hermetically sealed in a pouch cell. Cu–C was used to test the cathodic stability, and Al–C was used to test anodic stability; both were dried at 120 °C for 12 h under vacuum prior to assembly. Separate measurements were done to determine the cathodic and anodic stability limits, and all cells were stored at 40 °C for 72 h before starting the measurements.

### Reference Electrode Fabrication

**Figure 1:**
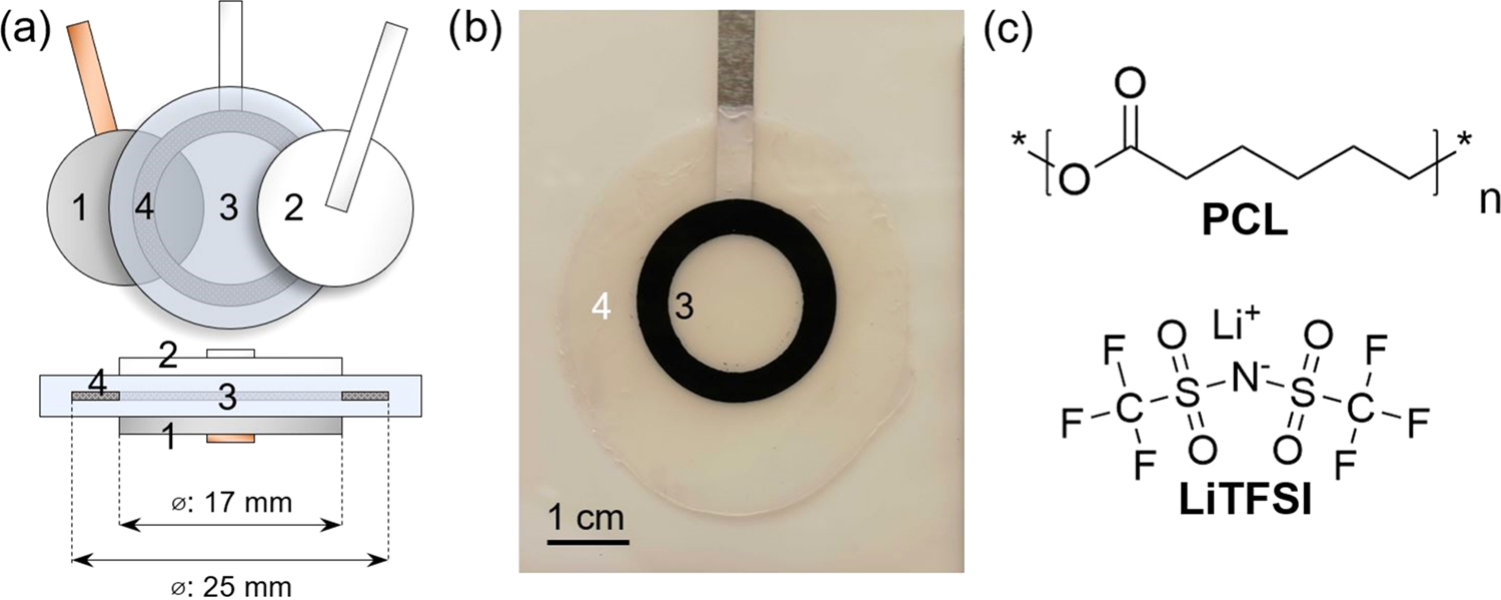
(a) Schematic of three-electrode cell consisting of lithium counter electrode (1), a carbon-coated aluminum or carbon-coated copper working electrode (2), PCL:LiTFSI solid polymer electrolyte (3), and a half-lithiated LiFePO_4_ reference electrode (4) embedded in the polymer electrolyte. (b) Photograph of the reference electrode embedded in the polymer electrolyte. (c) Molecular structure of poly(caprolactone) (PCL) and lithium bis(trifluoromethanesulfonyl)imide (LiTFSI) salt.

LFP reference electrodes were constructed by first cutting a ring (inner and outer diameters of 17 and 25 mm, respectively) out of a double-sided LFP coating with an areal capacity of 3.85 mAh cm^–2^. Next, the ring electrode was placed between two lithium metal counter electrodes, separated by two layers of a Celgard 2500 separator and 100 μL of the LP40 electrolyte on either side. The ring electrode and separators were dried under vacuum at 120 and 60 °C, respectively, for 24 h prior to assembly. The cell stack was hermetically sealed in a pouch cell and then galvanostatically delithiated at 270 μA for 20 h at room temperature using a Bio-Logic MPG2 (see Figure S2), whereupon the LFP was approximately 43% delithiated (assuming a specific capacity of 170 mAh g^–1^). Following delithiation, the cell was disassembled and the ring electrode was washed in DMC and dried under vacuum for 5 h at room temperature. Finally, the ring electrode was embedded in PCL:LiTFSI via hot pressing. This was achieved by first placing it between two sheets of PCL:LiTFSI in an MTI 6T hydraulic lamination hot press. The stack was preheated at 90 °C without any added pressure for the initial 30 min before pressing at 1 MPa for 30 min. Once 30 min had passed, the heater was turned off and the pressure was maintained until the temperature cooled to 40 °C. The reference electrode embedded in PCL:LiTFSI can be seen in Figure 1b.

### Staircase Voltammetry (SV)

The electrochemical stability window of PCL:LiTFSI was studied using staircase voltammetry (SV) combined with EIS using a Bio-Logic SP240 portable potentiostat at 40 °C. Three-electrode cells consisting of a lithium metal counter electrode, Cu–C or Al–C working electrode, and a PCL:LiTFSI polymer electrolyte with an embedded LFP reference were assembled and sealed in pouch cells. Next, the chronoamperometric response was measured as the potential was increased or decreased in 100 mV steps, each held for 1 h. Between each step, there was a 1 h pause after which the impedance–frequency response was measured between 10^6^ to 0.1 Hz with an amplitude of 10 mV followed by a second 10 min pause. The frequency range was limited to a maximum of 100 kHz for the counter electrode impedance measurements. Separate measurements were done to determine the cathodic and anodic stability limits, and all cells were stored at 40 °C for 72 h before SV measurements.

### X-ray Photoelectron Spectroscopy

X-ray photoelectron spectroscopy was done using a PHI 5500 hemispherical analyzer equipped with a monochromatic Al Kα (*h*ν: 1486.7 eV) photon source. The detector–sample stage angle was 45°, and the probing spot was approximately 1 × 1 mm with a probe depth of ca. 6–10 nm. Charging of nonconductive polymer samples was mitigated with the help of a neutralizer with a current of 20 mA at 20% energy. Peak fitting was done in CasaXPS v. 2.3.22PR1.0 using a 70% Gaussian/30% Lorentzian Voigt line shape mix and Shirley background subtraction. Energy calibration was applied according to the hydrocarbon peak at 285 eV belonging to the working electrode layer. The relative atomic composition (*C*_*i*_) was calculated using the atomic sensitivity factors reported in “The Handbook of X-ray Photoelectron Spectroscopy” and eq 1 ^26^
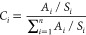
1where *A*_*i*_ is the area of the peak belonging to element *i* and *S*_*i*_ is the atomic sensitivity factor belonging to element *i*. The ratio is then divided by the total sum for all elements in the sample. The S 2p peaks were fitted according to a spin–orbit splitting of 1.18 eV. All spectra were normalized with respect to the highest intensity. Cells used for XPS were prepared in the same way as those used for voltammetry measurements. The working electrode potential was swept from open circuit voltage (OCV) to different cutoff potentials at 0.1 mV s^–1^ followed by a 3 h potential hold at 40 °C using a Bio-Logic VMP2 (see Figure 5). The cells were also cycled from OCV to −0.5 V and OCV to 5 V three times with the same scan rate and temperature. Once finished, the cells were dissembled and the polymer electrolyte surface adjacent to the working electrode and the working electrode were analyzed using XPS.

### Scanning Electron Microscopy (SEM)

Changes in surface morphology and elemental composition of the working electrodes and the polymer electrolyte postmortem were studied using a Carl Zeiss Merlin field emission scanning electron microscope equipped with energy-dispersive X-ray spectroscopy (EDS). Micrographs were taken with an acceleration voltage of 3 kV and a beam current of 100 pA. Elemental mapping was done using the same acceleration voltage but with a beam current of 1 nA. SEM samples were prepared from the same cells as those used for XPS.

### Lithium Plating and Stripping

The compatibility of PCL:LiTFSI with lithium metal was evaluated via lithium stripping and plating in combination with EIS. A three-electrode symmetrical cell consisting of two lithium electrodes (17 mm in diameter) and a PCL:LiTFSI polymer electrolyte film with an embedded LFP reference electrode was charged and discharged at 10 μA cm^–2^ for 3 h consecutively at 40 °C using a Bio-Logic SP240 portable potentiostat. Between each charge and discharge, the cell was allowed to rest for 1 h, after which the frequency–impedance response was measured between 10^6^ and 0.1 Hz with an amplitude of 10 mV followed by a second 10 min pause. The frequency range was limited to a maximum of 100 kHz for the counter electrode. The cell was kept at 40 °C for 72 h before lithium stripping and plating was started.

## Results and Discussion

The cathodic and anodic stability of PCL:LiTFSI was studied using staircase voltammetry (SV) at 40 °C. Unlike sweep voltammetry, holding the potential for an extended time not only ensures that the capacitive contribution to the current response is diminished but also reveals the stability of the electrolyte under static conditions.^18^ In addition, electrochemical impedance spectroscopy (EIS) was measured between each potential step to understand the implications of the current response on the resistance of the electrolyte–electrode interface. The impedance response originating from the working electrode was isolated using a three-electrode cell consisting of a lithium metal counter electrode, a carbon-coated working electrode, and a half-lithiated LiFePO_4_ (LFP) reference electrode; see Figure 1. We opted to use carbon-coated working electrodes to mimic the electrode surfaces found in LIBs. This is especially important since the electrode composition and morphology will affect the current response.^27^ The original purpose of the carbon coating is to create good electronic contact and adhesion between the electrode composite and current collector, and it has a negligible capacity contribution.^28,29^ The LFP reference was embedded in PCL:LiTFSI via hot pressing at 90 °C; see the Experimental Section for details. To guarantee that the polymer electrolyte did not chemically decompose during hot pressing, the thermal stability was evaluated using thermal gravimetric analysis (TGA). A weight loss of ∼80% was observed at 300 °C followed by an ∼10% loss at 360 °C, corresponding to the degradation of PCL and LiTFSI, respectively; see Figure S3a.^8,20^ However, when the temperature is increased in a series of steps, each held for 3 h, the onset of thermal degradation instead starts at 180 and 300 °C, respectively; see Figure S3b. It can also be concluded that the PCL:LiTFSI membrane was free from significant quantities of residual solvent.

**Figure 2:**
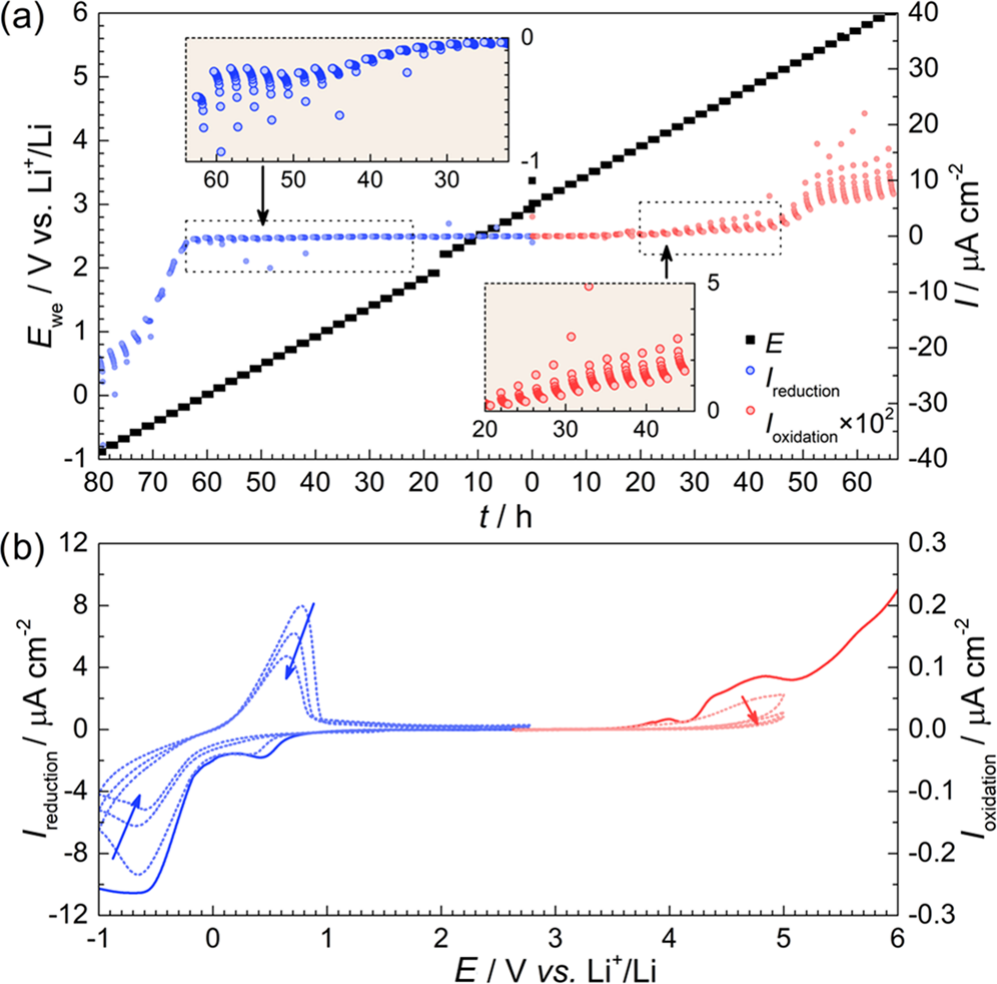
(a) Chronoamperometric response of PCL:LiTFSI at potential steps ranging from 3.0 to −1 V and 3.0–6.0 V vs Li^+^/Li at 40 °C. The oxidation current has been multiplied by 100 to improve legibility. (b) Linear sweep voltammetry (solid line) and cyclic voltammetry (dotted line) of Li | PCL:LiTFSI | Cu–C or Al–C with a scan rate of 0.1 mV s^–1^ at 40 °C.

The reduction and oxidation stability of PCL:LiTFSI from 3 to −1 V (in blue) and 3–6 V (in red) vs Li^+^/Li can be seen in Figure 2a. In the range of 3–1.5 V, no significant reduction current is observed, indicating limited degradation. Once below 1.5 V, the current response increases steadily with each step until 0.6 V, after which the current dips. The reduction onset at 1.5 V observed in this work agrees well with the reduction limit derived using atomistic modeling: 1.2 V vs Li^+^/Li for PCL:LiTFSI.^22^ The decrease in reduction current observed at 0.6 V occurs at a similar potential to the formation of H_2_ gas observed at 0.75 V vs Li^+^/Li in poly(trimethylene carbonate):LiTFSI.^30^ Hence, the decrease in current may be related to the reduction of trace amounts of H_2_O. Furthermore, the absence of the feature between 1.5 and 0.6 V during subsequent cycles indicates that a passivation reaction took place, or that all of the reactants were consumed (see Figure 2b). At potentials below 0 V, the reduction current increases substantially as lithium plating on the working electrode commences. From 0 to −0.5 V, the current increases during each potential step which could be due to an initial nucleation process when lithium is deposited.^31^ Once the nucleation sites reach a critical size, the current behavior returns to normal as seen below −0.5 V. The overpotential for lithium plating is also observed in the two subsequent cycles, indicating that the nucleation sites have to reform each time (see Figure 2b).

At the start of the anodic sweep, the oxidation current is null; see the red dots in Figure 2a. From 4 V and onward, the oxidation current increases until the potential step at 4.5 V is reached, after which it stabilizes. In PTMC:LiTFSI, SO_2_ formation has been observed at 4.4 V, indicating LiTFSI degradation.^30^ Similar degradation mechanisms can be expected in PCL:LiTFSI and give rise to the current feature between 4 and 5 V. However, it should be noted that Marchiori et al. estimated the upper ESW boundary of PCL:LiTFSI to 5.4 V vs Li^+^/Li, far higher than that observed here.^22^ In comparison, PEO:LiTFSI exhibited an oxidation onset at approximately 3.5 vs Li^+^/Li using SV at 60 °C.^18^ According to cyclic voltammetry, the current response observed between 4 ad 5 V decreases with each sweep, indicating either a gradual consumption of reactants or passivation at the electrolyte–electrode interface, which prevents further electrochemical degradation; see Figure 2b. At 5 V, the oxidation current increases once again. At such high potentials, localized corrosion pitting of the protective oxide on the aluminum current collector in the presence of the TFSI anion can be expected.^32,33^

**Figure 3:**
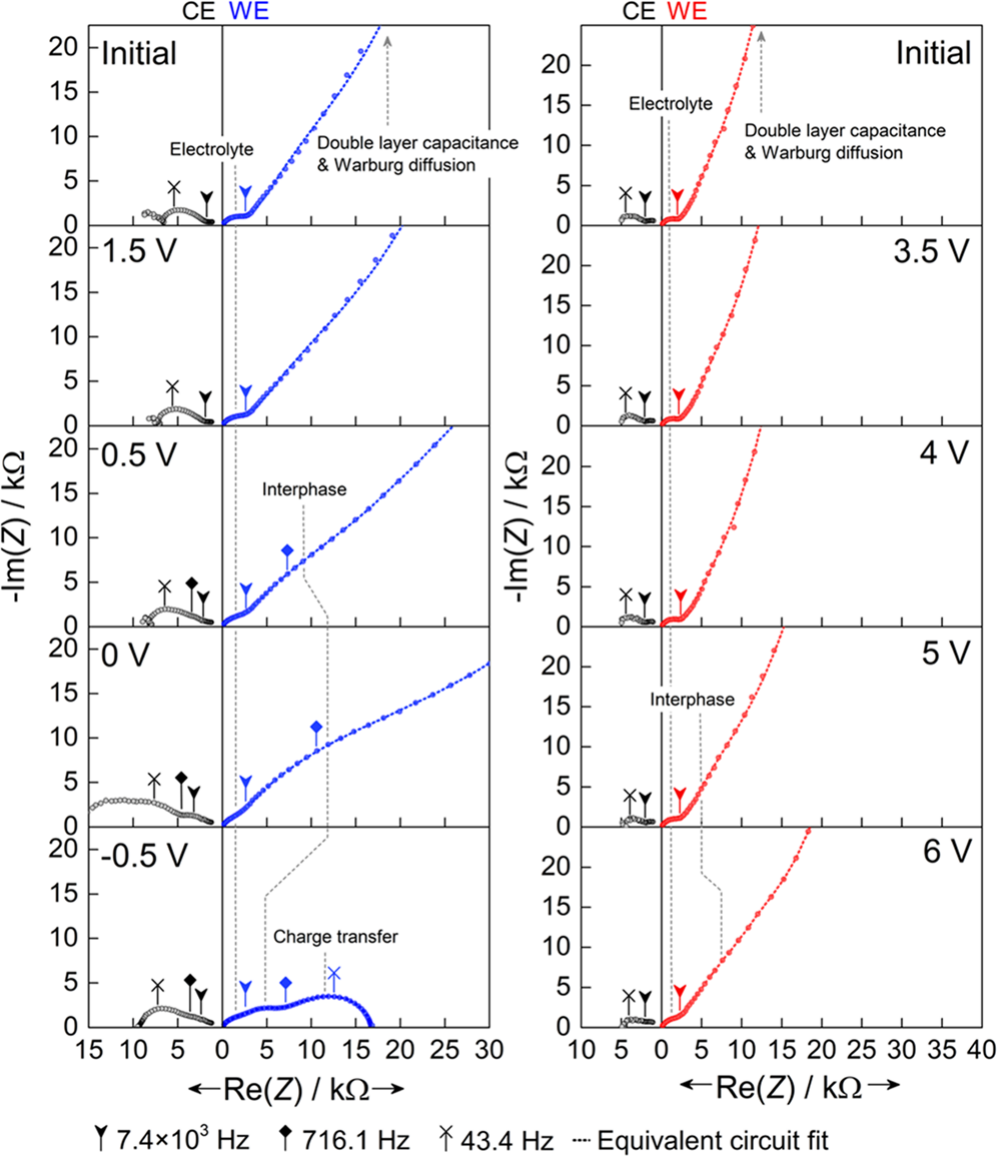
Impedance response between the working electrode (WE) and reference electrode after each potential step at 40 °C. The impedance response between the counter electrode (CE) and the reference electrode at 40 °C is shown in light gray.

Based on the chronoamperometric response, the frequency–impedance response of the working electrode was studied in detail at specific potentials: start of the measurements (initial); before the onset of reduction and oxidation (1.5 V and 3.5 V, respectively); before, during, and after lithium plating (0.5, 0, and −0.5 V); and at higher potentials (4, 5, and 6 V) (see Figure 3). The complete set of Nyquist plots can be seen in Figure S4. Initially, a single depressed semicircle at high frequencies (*f* > 10^3^ Hz) followed by a tail at lower frequencies (*f* < 10^3^ Hz) is observed, typically associated with ion transport in the bulk polymer electrolyte and a combination of double-layer capacitance and diffusion processes, respectively.^7^ The same impedance response is observed when PCL:LiTFSI is placed between two stainless steel blocking electrodes (see Figure S5a). Hence, the carbon-coated metal foils behave as blocking electrodes at the start of the experiment. In agreement with the chronoamperometric measurement, these features remain unchanged at 1.5 and 3.5 V. At 0.5 V, a partial protuberance located between the semicircle and the tail becomes visible. This feature originates from a highly resistive interphase at the working electrode–polymer interface.^15^ At 0 V, the feature has grown more prominent, suggesting either further interphase growth or compositional alteration. Below 0 V, the highly resistive interphase feature is replaced by two semicircles with lower overall resistance. The semicircles located at mid (10^3^–10^2^ Hz) and low (10^2^–0.1 Hz) frequencies belong to the lithium–electrolyte interphase and the charge transfer resistance, respectively,^16^ and share resemblance to the impedance response observed between the lithium counter electrode and the LFP reference electrode; see Figure S4. Furthermore, a similar impedance silhouette was also seen during stripping and plating in a Li | PCL:LiTFSI symmetrical cell; see Figure S6. In contrast, only minor changes were observed going to higher potentials, with a barely visible partial semicircle appearing at 5 V. This is not surprising seeing that the reduction current is approximately 10-fold larger in magnitude (prior to plating) compared to the oxidation current.

**Figure 4:**
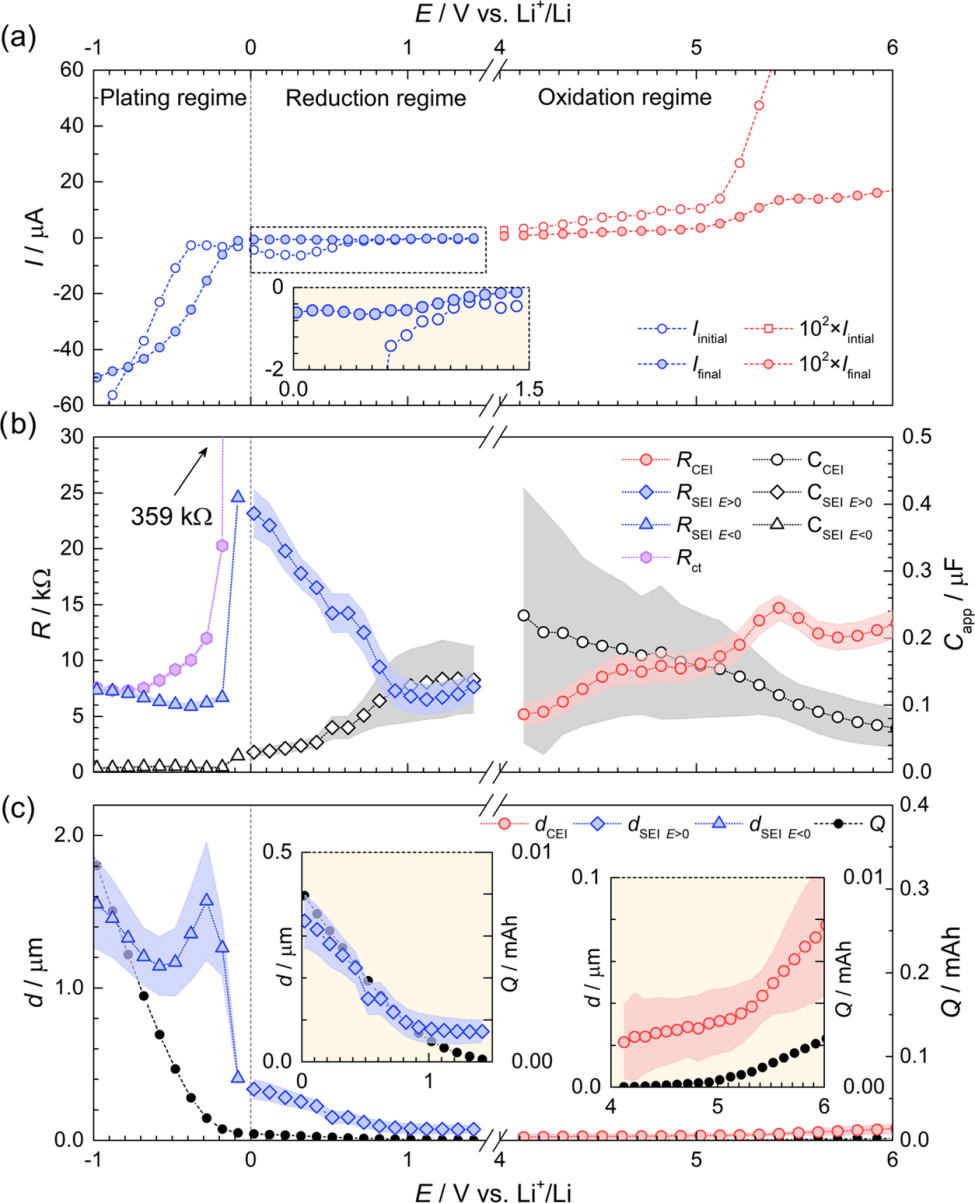
(a) Initial and final relaxation currents of each potential step at 40 °C. (b) Resistance and apparent capacitance of cathode electrolyte interphase (*R*_CEI_ and *C*_CEI_), solid electrolyte interphase above 0 V vs Li^+^/Li (*R*_SEI *E*>0_ and *C*_SEI *E*>0_), solid electrolyte interphase below 0 V (*R*_SEI *E*<0_ and *C*_SEI *E*<0_), and charge transfer resistance (*R*_ct_) between each potential step in the SV measurement at 40 °C. The shaded region represents equivalent circuit fitting errors. (c) Estimated thickness of cathode electrolyte interphase (*d*_CEI_), solid electrolyte interphase above 0 V (*d*_SEI *E*>0_) and below 0 V (*d*_SEI *E*<0_) based on eq 3 (in color), and accumulated redox charge (*Q*). The shaded regions represent propagated errors.

For reference, the initial and final relaxation current values for each potential step versus potential can be seen in Figure 4a. The impedance response between 1.5–0 and 4.1–6 V vs Li^+^/Li—corresponding to SEI and cathodic electrolyte interface (CEI) formation—was modeled using circuit A; see Table S1. From 0 to −1 V, lithium plating and additional SEI formation on the working electrode were modeled using circuit B.^16^ The goodness of fit (χ^2^) ranged from 10^–4^ to 10^–5^. Both circuits A and B are simplified representations of the processes occurring at the electrode–electrolyte interface and the bulk electrolyte. A Warburg element was omitted since the impedance measurement did not go to sufficiently low frequencies to resolve the contribution from diffusion. Changes in interphase resistance and capacitance during cathodic and anodic degradation were extracted via equivalent circuit fitting and can be seen in Figure 4b. Capacitances were modeled using constant phase elements (CPE) instead of capacitors to consider surface roughness, nonuniform current density, and varying reaction rates. Consequently, the apparent capacitance (*C*_app_) was calculated using the following equation (given *n* > 0.75)^16^
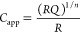
2where *R* is the resistance, *Q* is the CPE capacitance, and *n* is the phase angle. The calculated apparent capacitances and interphase resistance values obtained using equivalent circuit fitting can be seen in Figure 4b. As seen in Figure 4b, *R*_CEI_ and *R*_SEI *E*>0_ are inversely correlated to *C*_CEI_ and *C*_SEI *E*>0_, respectively. Between 4.1 and 5.4 V, the CEI interphase resistance (*R*_CEI_) triples from approximately 5 to 15 kΩ and then decreases to 13 kΩ. From 1.5 to 1 V, the SEI interphase resistance (*R*_SEI *E*>0_) remains constant at 7 kΩ, after which it rapidly increases to 23 kΩ accompanied by a decrease in *C*_SEI *E*>0_. A similar trend was observed at the interface between Cu and a glyme-based solvate ionic liquid with LiTFSI at 0 V, attributed to the formation of a low-conductivity SEI layer.^34^ In comparison, the resistance of the SPE is only ∼2.5 kΩ, despite having a thickness of ∼125 μm. Below 0 V, the interphase resistance (*R*_SEI *E*<0_) drops rapidly and stabilizes at 7 kΩ from −0.2 to −1 V. In conjunction, the charge transfer resistance (*R*_ct_) also decreases, suggesting an initial kinetic barrier for lithium deposition.

The thickness (*d*) of the CEI and SEI at different potentials (see Figure 4b) was calculated based on *C*_app_ using the following equation^35^
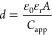
3where ε_0_ is the permittivity of free space, ε_r_ is the relative permittivity of the interphase, and *A* is the area of the interphase. Based on the literature, the relative permittivity of the CEI and SEI is assumed to be 5 and 10, respectively.^16,35−37^ It is estimated that the interphase covers approximately 50% of the working electrode on average, based on the poor electrode–electrolyte contact intended to facilitate disassembly for postmortem analysis. However, the electrode area is probably not constant during the measurement, especially during lithium plating, and as such can lead to an underestimation of the interphase thickness. Hence, an alternative strategy to determine the surface area in operando should be devised in the future for more precise results.

As seen in Figure 4b, the thickness of the CEI increases gradually from 21 ± 18 to 82 ± 36 nm between 4.1 and 6 V. In comparison, the interphase between PEO:LiCF_3_SO_3_ and a V_6_O_13_ cathode reportedly increased from 15 to 75 nm over a period of 200 h at 100 °C.^37^ Similarly, the thickness of the SEI increases from 73 ± 26 to 336 ± 63 nm between 1.5 and 0 V. In comparison, the calculated thickness for the interphase between lithium metal and PEO:LiClO_4_ was 12 nm after passive contact for 1 week.^35^ Nonetheless, 336 ± 63 nm is relatively thick considering the minimal convection expected in SPEs and would only be possible if the interphase species was partially electronically conductive. In fact, linear scaling density functional theory calculations have shown that with the addition of LiTFSI in the polymer host, the band gap of the SPE is significantly reduced, resulting in a lower threshold for electronic leakage currents.^38^ Alternatively, the formation of oligomeric species following polymer chain scission could increase the convection of degradation species, thereby allowing further decomposition. As seen in Figure 4c, the magnitudes of *d*_CEI_ and *d*_SEI *E*>0_ correlate with the cumulative charge (*Q*) (e.g., integration of current over time), suggesting that the cathodic and anodic currents seen in Figure 4a originate from electrochemical degradation and subsequent interphase formation.

Counterintuitively, the SEI thickness obtained in this work is thicker compared to that typically observed in liquid electrolyte systems, where the initial thickness of the SEI estimated using XPS typically falls in the range of 20 nm and grows with subsequent cycling.^39^ However, it is worth noting that the thickness of the SEI in liquid electrolyte systems only represents the solid phase that is present on the anode after it has been extracted from the battery and placed under high vacuum conditions in preparation for postmortem XPS or SEM, where all liquid components are no longer present. Using other techniques, e.g., EQCM-D, results indicate the presence of a much thicker region of compounds, in the range of 100 nm already in the first cycle, that to some extent is adsorbed/attached to the anode surface.^40^

Below 0 V, the thickness of the interphase increases rapidly from 407 ± 35 nm to 1.68 ± 0.37 μm following the onset of lithium plating. According to CV, the first reduction sweep to −1 V had a coulombic efficiency of 39%, followed by 43 and 49% in the following cycles; see Figure 2b. Finally, the average interphase ionic conductivity (σ) was calculated using the following equation
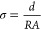
4where *R* is the interphase resistance, *A* is the interphase area, and *d* is the interphase thickness. According to eq 4, the average ionic conductivities of the CEI, SEI at *E* > 0, and SEI at *E* < 0 were 3.5 ± 0.2 × 10^–9^, 1.0 ± 0.3 × 10^–9^, and 1.4 ± 0.3 × 10^–8^ S cm^–1^, respectively. The ionic conductivity of the SEI at *E* < 0 is similar to the conductivity previously reported for the stabilized interphase between lithium metal and PEO:LiTFSI (3 ± 1 × 10^–8^ S cm^–1^) at 90 °C.^16^ It has been shown that the ionic conductivity of the interphase between lithium metal and SPEs exhibits temperature behavior synonymous with Arrhenius-like transport.^35^ Switching from one mode of transport in the bulk electrolyte to another in the interphase could have implications on interfacial ionic resistance. Relative to the polymer electrolyte conductivity at 40 °C (2.6 × 10^–6^ S cm^–1^, see Figure S5b), the CEI and SEI represent major bottlenecks in the polymer electrolyte system.

**Figure 5:**
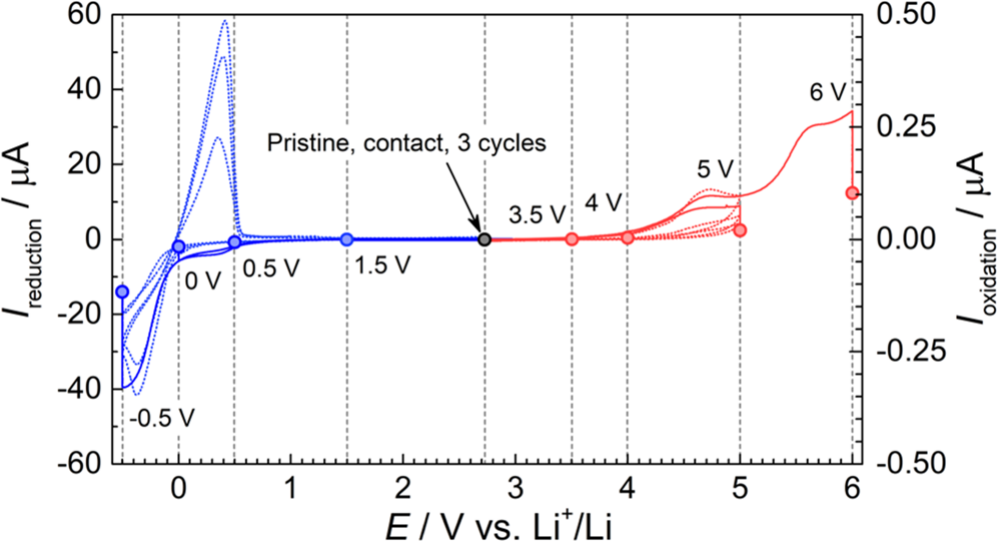
Current profiles of Li | PCL:LiTFSI | Cu–C or Al–C cells taken apart for postmortem morphological and compositional analysis. Current response during linear sweep to different cutoff potentials followed by a potential hold for 3 h (solid line) and during the first three cycles (dotted line). All measurements were done with a scan rate of 0.1 mV s^–1^ up to the cutoff potential at 40 °C.

Samples for postmortem morphological and compositional analysis were prepared by cycling two-electrode cells, consisting of a lithium metal counter electrode and a carbon-coated working electrode, from OCV (∼2.7 V vs Li^+^/Li) to different potential cutoffs followed by a 3 h potential hold to amplify interphase formation; see Figure 5. In addition, cells were cycled from OCV to −0.5 and OCV to 5 V three times to distinguish the buildup of irreversible interfacial species during reduction and oxidation, respectively; see Figure 5. Separating the electrolyte–electrode interface for postmortem analysis, while keeping interphases intact, is a difficult task due to the adhesive property of polymer electrolytes.^15,41^ To this end, four strategies were employed to facilitate separation of the layers, albeit at the expense of optimal cycling conditions:^42^ (1) cycling was carried out below the *T*_m_ of PCL:LiTFSI (43.6 °C, see Figure S7) to prevent the electrolyte from sticking to the electrode, (2) the salt concentration was limited to 20 wt % to ensure mechanical robustness, (3) the polymer electrolyte was cast separately to prevent extensive infiltration, and (4) minimal stack pressure was applied. These strategies were implemented for all samples throughout this work.

**Figure 6:**
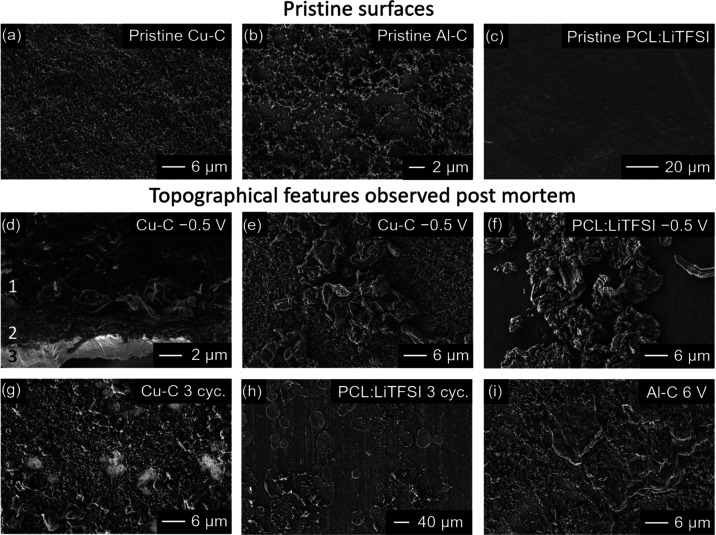
SEM micrographs of (a) pristine Cu–C electrode; (b) pristine Al–C electrode; (c) pristine PCL:LiTFSI membrane; (d) cross section of Cu–C electrode from an angle showing layering of (1) polymeric layer, carbon coating (2), and Cu current collector (3); (e) dendrite-like structures on top of Cu–C; (f) dendrite-like structures attached to the polymer electrolyte adjacent to Cu–C electrode and (g) Cu–C electrode after three cycles; (h) circular patterns left in the absence of dendrite structures; and (i) polymeric species on top of the Al–C electrode.

The surface topography of the carbon-coated copper and aluminum working electrodes, hereafter referred to as Cu–C and Al–C, respectively, and PCL:LiTFSI at different potential stages was studied using scanning electron microscopy (SEM) and energy-dispersive X-ray spectroscopy (EDS); see Figures 6 and S8. As seen in Figure 6a,b, the pristine surface of Cu–C and Al–C is covered in a thin layer of conductive carbon particle agglomerates. In contrast, the pristine PCL:LiTFSI membrane has a smooth surface; see Figure 6c. Following reduction to −0.5 V vs Li^+^/Li, two different types of interfacial morphologies were observed at the Cu–C electrode surface. The first layer can be seen in Figure 6d (marked with 1), which appears to be polymeric and was easily damaged by the electron beam. The second layer consists of dendrite-like structures approximately 1 to 5 μm in size; see Figure 6e. These structures also covered a large portion of the PCL:LiTFSI surface (Figures 6f and S8a) and are rich in oxygen (see Figure S8b). Based on the EDS images, it is difficult to tell if the core of the structures consists of something other than oxide species, e.g., plated lithium. The sites on the Cu–C and PCL:LiTFSI surfaces that did not show these features were similar to the pristine sample. Interestingly, the dendrite structures share little resemblance with the mossy dendrites observed on the surface of lithium following 17 stripping and plating cycles (see Figure S9). Following three plating and stripping cycles from OCV to −0.5 V vs Li^+^/Li, these dendrite-like structures are stripped from the Cu–C surface. Left behind are small inorganic residues and exposed copper foil (see Figures 6g and S8c,d). SEM imaging of the adjacent polymer surface revealed circular patterns in place of the dendrite structures (see Figure 6h). Upon closer inspection, these circular patterns appear to be perforations in the electrolyte membrane that are filled with small granules, presumable irreversibly formed oxide species, or dead lithium that has lost electronic contact (see Figure S8e–h). Based on this observation, it appears that the polymer electrolyte membrane has been pierced by these dendrites. If the polymer electrolyte membrane is too thin, severe dendrite growth can cause erratic potential behavior and eventually short-circuit the cell.^42,43^ This may explain why a Li | PCL:LiTFSI | LiFePO_4_ cell was unable to cycle for more than 16 cycles at 60 °C (bearing in mind that the molecular weight was much lower in comparison to this work).^24^ Furthermore, as demonstrated by Bergfelt et al., the cell life span was prolonged by incorporating a polystyrene block, thereby increasing the membrane’s mechanical stability.^24^ Dendrite growth can also decrease the interelectrode distance, which results in a lower internal resistance;^42^ however, this phenomenon was not observed here. In contrast, only small polymeric residues were observed on the Al–C electrode following oxidation to 6 V vs Li^+^/Li (see Figure 6i). In addition, a few carbon particles were observed on the electrolyte membrane, most likely carbon black that delaminated from the Al–C electrode (see Figure S8i,j).

**Figure 7:**
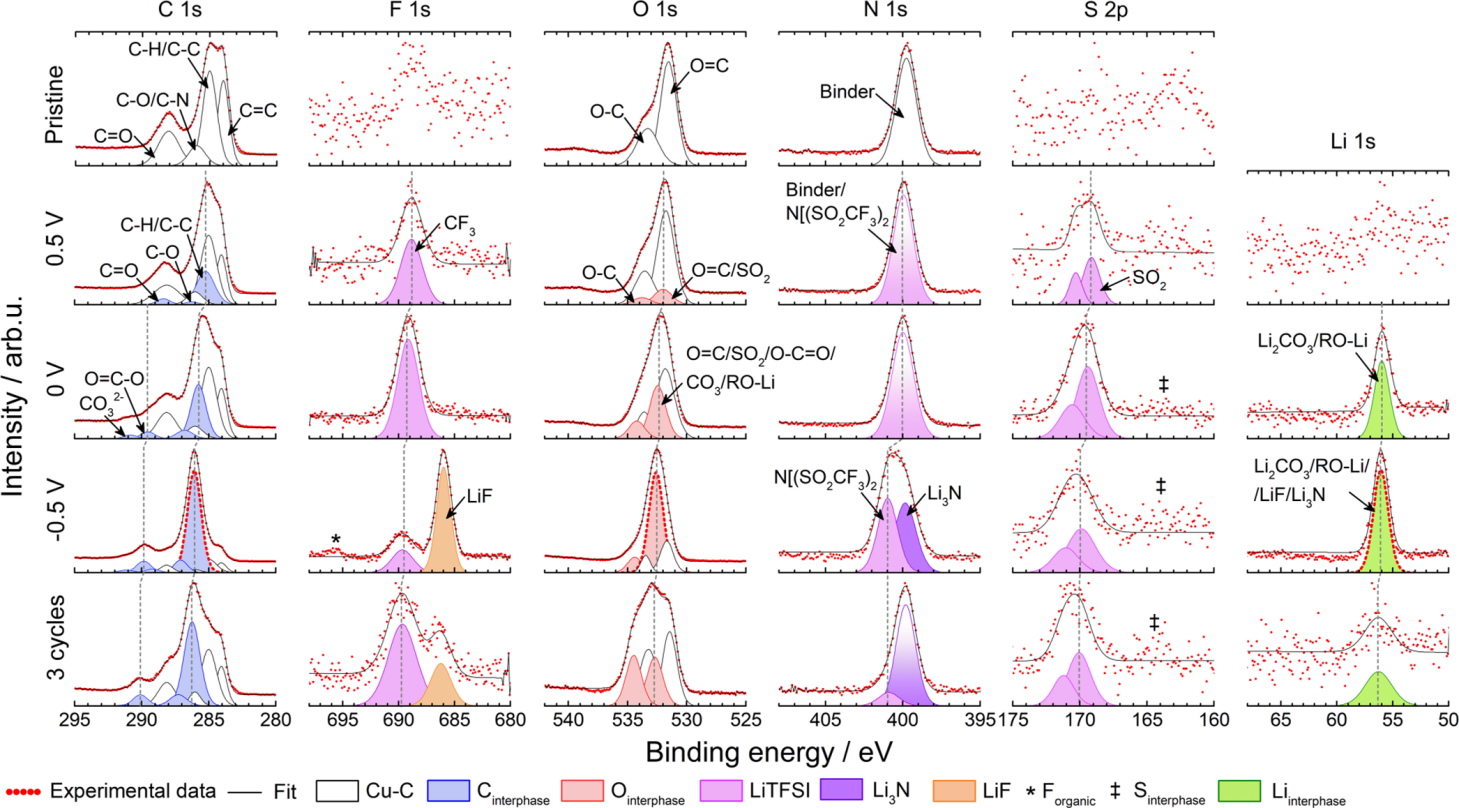
XPS spectra from the Cu–C surface at different stages: prior to assembly (pristine), at 0.5, 0, and −0.5 V vs Li^+^/Li and after three cycles from OCV to −0.5 V. Spectra were normalized according to the highest intensity count in each spectrum.

Compositional changes at the electrolyte–electrode interface during reduction and oxidation were analyzed using postmortem XPS. The spectra pertaining to the Cu–C electrode and PCL:LiTFSI surfaces prior to assembly (pristine), following contact at 40 °C for 72 h, after reduction to 0.5, 0, and −0.5 V, and after three cycles from 2.7 to −0.5 V can be seen in Figure 7. Complementary spectra taken after contact and cycling to 1.5 V showed almost identical spectra as the pristine sample and can, for reference, be seen in Figures S10 and S11, respectively. XPS fitting was primarily done by superposition of the SPE profile onto the pristine electrode profile, followed by the addition of peaks belonging to relevant decomposition compounds. The XPS spectra were calibrated versus a bulk component (C–C peak placed at 285 eV). It is thus expected that peaks related to electronically conductive parts should remain at a constant binding energy position while small but similar shifts in peak position are expected for nonelectronically conductive parts (here the SPE) for samples with different cutoff potentials.^44^ For example, at 0.5 V, this shift is equivalent to ∼0.2 eV, and as the cutoff potential decreases, this shift is expected to increase; see the vertical dotted lines in Figure 7. XPS of the pristine Cu–C electrode reveals C 1s peaks at 284.0, 285.0, 286.0, and 288.1 eV corresponding to C=C, C–C/C–H, C–O/C–N, and C=O, indicating carbon black and possibly adventitious carbon on the copper surface. According to the manufacturer, the carbon black is held together using an organic binder, most likely containing nitrogen as indicated by the peak at 399.8 eV.^28,29^ Two Cu 2p peaks are also observed at 932.7 and 934.3 eV, corresponding to Cu and copper oxides; see Figure S10. As seen in Figure S10, the Cu–C electrode interface remained more or less unchanged after contact for 72 h at 40 °C and after reduction to 1.5 V vs Li^+^/Li. In Figure 7, additional peaks at 285, 286, and 288.2 eV in the C 1s spectrum (in blue), corresponding to C–C/C–H, C–O/C–N, and C=O, indicate the presence of carbonaceous species on top of the Cu–C electrode at 0.5 V. The presence of C=O indicates breaking of the C–O ester bond in PCL, as suggested by Ebadi et al.^23^ The carbon peaks are accompanied by two additional O 1s peaks at 532 and 533.8 eV (marked in red), corresponding to C=O and C–O. Furthermore, the presence of a spin–orbit split S 2p peak at ∼170 eV (in purple) and an F 1s peak at 688.8 eV (in purple), corresponding to S=O and CF_3_, indicates the presence of TFSI^–^ (intact) on the surface; see Figure 7.^45,46^ The N 1s peak at 400 eV, corresponding to nitrogen in the TFSI^–^, overlapped with the nitrogen peak from the binder; thus, identification of the salt is not possible in the N 1s spectra.^41^ However, the intensity of the N 1s peak relative to the C=C peak increases from approximately 0.5 to 0.7 in the pristine sample and the 0.5 V sample, respectively, demonstrating that TFSI^–^ also contributes to the N 1s intensity; see Figure S12.

**Figure 8:**
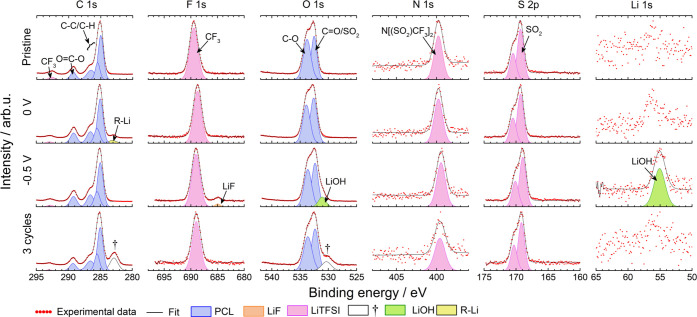
XPS spectra from the PCL:LiTFSI surface adjacent to the Cu–C electrode at different stages: prior to assembly (pristine), at 0 and −0.5 V vs Li+/Li and after three cycles from OCV to −0.5 V. Spectra were normalized according to the highest intensity count in each spectrum.

At 0 V vs Li^+^/Li, the C 1s peaks belonging to the organic interphase species (marked in blue), with the exception of C=O, increased in intensity relative to the peaks corresponding to the Cu–C working electrode (marked in white), indicating a buildup of small amounts of carbonaceous species at the interface. Interestingly, the absence of C=O suggests that the C_carbonyl_–O_ester_ bond remained intact. Furthermore, two new C 1s peaks were also observed at 289.5 and 290.8 eV, corresponding to O=C–O, and CO_3_^2–^. The O=C–O peak position matches well with the O=C–O peak belonging to PCL; see Figure 8. Furthermore, the F 1s and S 2p peaks (in purple) increased in intensity relative to the carbonaceous interphase peaks (in blue), indicating an accumulation of TFSI^–^ at the interface. However, according to the relative atomic composition, the F 1s peak constitutes a small portion of the interphase (1.6%, see Figure S12), which explains why no CF_3_ peak was observed in the C 1s spectra. In addition, there appears to be a feature at ∼164 eV (marked with ‡), possibly indicating the presence of polysulfides or Li_2_S.^21^ This feature becomes slightly more visible after three cycles, but still constitutes a very small portion of the interface; see Figure 7. A new Li 1s peak is also observed at 56 eV (marked in green); see Figure 7. Possible candidates for this peak include Li_2_CO_3_, LiOH, RO–Li, LiF, Li_3_N, and Li_2_S.^21,41,47,48^ However, the absence of matching peaks in the O 1s, F 1s, N 1s, and S 2p spectra at 0 V rules out LiOH, LiF, Li_3_N, and Li_2_S. In agreement with this work, no LiF was observed at the interface between triglyme:LiTFSI solvate ionic liquid and Cu at 0 V, indicating minimal TFSI^–^ degradation.^34^ According to the relative atomic composition, the concentration of lithium species is approximately 5 times larger than the CO_3_^2–^ concentration; see Figure S12. Hence, it can be concluded that the Li 1s peak does not exclusively belong to Li_2_CO_3_. Furthermore, Li_2_CO_3_ formation is typically observed in the presence of CO_2_ and OH^–^.^49,50^ However, previous modeling studies have suggested that CO_2_ is an unlikely degradation product of PCL.^23^ This is supported by the nonprominent CO_3_^2–^ peak in the C 1s spectra. The O 1s peak could also correspond to RO–Li, following the breaking of the C_carbonyl_–O_ester_ bond in the presence of lithium metal.^21,23,48,51^ To summarize, the Li 1s peak corresponds to a mix of Li_2_CO_3_ and RO–Li at 0 V.

At −0.5 V vs Li^+^/Li, a large portion of the Cu–C interface consists of organic and lithium interphase species. In agreement, an interphase consisting largely of hydrocarbons and lithium species was also reported for the interface between graphite and PEO:LiTFSI.^41^ In conjunction with interphase growth between 1.5 and −0.5 V, the two Cu 2p peaks at 932.7 and 934.3 eV became less and less distinguishable and visible, indicating that the Cu–C electrode is gradually covered; see Figure S10. Coinciding with the large increase of the Li 1s peak, a large increase of the C 1s and O 1s peaks at 286.1 and 532.6 eV, respectively, is also observed (marked in a thick red dotted line). Based on the stoichiometric relationship between these peaks, this suggests additional RO–Li formation.^52^ In agreement with the cyclic voltammogram, the intensity of the Li 1s peak is significantly lower following three cycles of plating and stripping, indicating a certain degree of reversibility. For the −0.5 V sample, a new F 1s peak is observed at 686 eV (in orange), corresponding to LiF, which is a byproduct of TFSI^–^ degradation.^21,41^ The relative intensity between the LiF and the CF_3_ peak in the F 1s spectrum clearly shows that a majority of the TFSI anions are decomposed at the interface. In addition, a new unidentified feature was observed at 695.8 eV (marked with *). Another telltale sign of TFSI^–^ decomposition was also observed at 399.8 eV in the N 1s spectra (dark purple color), corresponding to Li_3_N.^21,41^ Alternatively, this peak could belong to the binder; however, a peak of this magnitude seems improbable given the low intensity of the Cu–C peaks (in white) relative to the interphase peaks in the C 1s spectrum.

The XPS profile of pristine PCL:LiTFSI consists of a series of well-defined peaks; see Figure 8. C 1s peaks are observed at 285, 285.5, 286.5, and 289.3 eV (marked in blue), corresponding to C–H, C–H, C–H, and O–C=O in the polymer.^48,53^ The two different oxygen environments are also observed in the O 1s spectra at 532.5 and 533.8 eV (marked in blue).^53^ It should be noted that a portion of the O 1s peak at 532.5 also belongs to S=O in TFSI^–^.^45,46,48^ Peaks belonging to TFSI^–^ are also observed at 292.7, 689.5, 399.8, and ∼170 eV (marked in purple) in the C 1s, F 1s, N 1s, and S 2p spectra, respectively. No changes in interfacial composition were observed at the PCL:LiTFSI surface following contact and after reduction to 1.5 and 0.5 V vs Li^+^/Li; see Figure S11. At 0 V, a C 1s peak at 283.1 eV (marked in yellow) was observed, conceivably corresponding to R–Li that has dislodged from the electrode.^21^ At −0.5 V, two peaks at 55.1 and 531.1 eV (marked in green) are observed in the Li 1s and O 1s spectra, respectively. In addition to lithium oxide species, small quantities of LiF were also observed at −0.5 V; see Figure 8. The peak at 531.1 eV can be assigned to either LiOH or RO–Li (Li_2_O is typically observed at ∼528 eV).^21,47,48,52^ However, EDS imaging of the PCL:LiTFSI surface revealed that the dendritic structures consist mainly of oxygen, thus ruling out RO–Li; see Figure S8b. A significant LiOH formation is somewhat unexpected. The water content for PTMC:LiTFSI prepared using the same casting and drying method used herein has been reported to be less than 40 ppm.^21^ However, it has been demonstrated that LiTFSI is prone to degradation in the presence of H_2_O in ionic liquid systems, which agrees with the observed LiOH, LiF, and Li_3_N formation.^54,55^ Again, no discernible peak belonging to CO_3_^2–^ was observed in the C 1s spectra, hence ruling out the presence of Li_2_CO_3_. A plausible explanation as to why the R–Li peak is no longer visible at −0.5 V is that it is either covered by newly deposited interfacial species or R–Li is still attached to the adjacent Cu–C surface. When the lithium is stripped from the interface, two peaks at 282.9 and 530.3 eV (marked by †) are observed in the C 1s and O 1s spectra, respectively. At first glance, the peaks can be assigned to R–Li and RO–Li; however, the absence of a prominent peak in the Li 1s spectrum suggests that they correspond to something else. Based on the EDS mapping, the O 1s peak at 530.3 eV could belong to copper oxide species; see Figure S8e–h. In addition, if the carbon coating has delaminated from the Cu current collector, then it could give rise to the peak observed at 282.9 eV in the C 1s spectra.

XPS spectra of the Al–C electrode and PCL:LiTFSI surfaces prior to assembly (pristine), contact, after oxidation to 3.5, 4, 5, and 6 V, and after three cycles from 2.7 to 5 V can be seen in Figures S14 and S15, respectively. In contrast to the electrolyte–electrode interface during reduction, no significant changes were observed on the carbon-coated aluminum electrode (Al–C) and PCL:LiTFSI surface; see Figures S14 and S15, respectively. Without going into too much detail, C 1s peaks at 285.8, 287.4, 289.3, and 291.2 eV (marked in blue), corresponding to C–C/C–H, C–O/C–N, C=O, and CO_3_^2–^ were observed following contact and after oxidation; see Figure S14. Matching peaks were also observed in the O 1s spectra at 533.9, 534.9, and 535.7 eV, corresponding to CO_3_^2–^, C=O, and C–O, respectively. In addition, C 1s, F 1s, N 1s, and S 2p peaks, synonymous with TFSI^–^, were observed at 294.1, 690.5, 401.2, and ∼170 eV (marked in purple), respectively. The only indication of salt degradation is the presence of a C 1s peak at 292.6 eV (highlighted in yellow), corresponding to CF_2_ fragments, and a feature in the S 2p spectra between 163 and 166 eV (marked with ‡). In contrast to the SEM image in Figure 6b, XPS analysis of pristine Al–C shows that the aluminum current collector is covered in carbon and binder. An unidentified F 1s peak at 686.8 eV (marked with *) was briefly observed at 6 V, perhaps corresponding to traces of AlF_3_, suggesting aluminum corrosion.^56,57^ Two features were also observed at 121 and 118 eV in the Al 2s spectra at 6 V, corresponding to AlF_3_ and Al_2_O_3_, respectively.^56^ A portion of the 121 eV peak may also belong to Al-TFSI, also indicating aluminum corrosion.^57^

All in all, this depicts an ESW spanning from 1.5 to 4 V vs Li^+^/Li versus a carbon-coated working electrode at 40 °C. The SV data are corroborated by the negligible changes in interface composition observed using XPS. Outside of this potential range, PCL:LiTFSI undergoes electrochemical degradation. Between 1.5 and 0.5 V, PCL:LiTFSI degrades to form an SEI consisting predominantly of polymer-derived species and traces of intact TFSI^–^. Going from 0.5 to 0 V, the interphase resistance rapidly increases, accompanied by the formation of lithium alkoxide and carbonate species at the interface. As exemplified by the absence of this current peak in subsequent CV cycles, the blend of interphase species passivates the electrode surface. Based on the impedance response, we estimate the thickness of the SEI to be approximately 340 nm. Notably, no salt degradation species were observed in this potential range.

At −0.5 V, the interface consists primarily of polymeric and lithium alkoxide species and traces of LiF and Li_3_N. Despite cyclic voltammetry clearly showing partially reversible lithium plating peak, lithium metal was not observed using XPS. This may be expected based on the reactivity of lithium combined with the surface sensitivity of XPS.^48^ EDS mapping of the dendritic structures on the polymer electrolyte surface reveals them to be rich in oxygen, most likely corresponding to LiOH stemming from water impurities. From an industrial perspective, the presence of H_2_O impurities may be unavoidable since most large-scale cell assembly is conducted in dry rooms with relatively high moisture levels in comparison to gloveboxes. Nevertheless, following the onset of lithium plating, the interphase resistance dropped rapidly. Based on SEM micrographs, we attribute this behavior to the formation of lithium dendrites, which penetrate the existing SEI, providing an alternative pathway with less resistance. This is also reflected in the initial behavior of the charge transfer resistance, which is initially big but decreases rapidly and eventually plateaus. At this point, we estimate the thickness of the interphase (SEI and lithium dendrites) to be approximately 1.7 μm based on impedance measurements. The formation of dendrites does not appear to be uniform. Following three consecutive cycles, these structures are effectively stripped from the surface leaving behind perforations in the electrolyte filled with small inorganic particles. XPS analysis of the polymer electrolyte surface also revealed large quantities of unidentified species. It can thus be concluded that the electrolyte continues to degrade when in contact with deposited lithium dendrites. Future efforts should be devoted to achieving uniform lithium plating “behind” the SEI to prevent further polymer electrolyte degradation and achieve higher plating and stripping coulombic efficiencies. Ideally, this would be achieved by creating a uniform SEI with lower resistance.^34,58,59^ Alternatively, one could use a polymer electrolyte that is stable toward lithium metal, thereby circumventing the need for an SEI altogether, provided that such a material exists.

**Figure 9:**
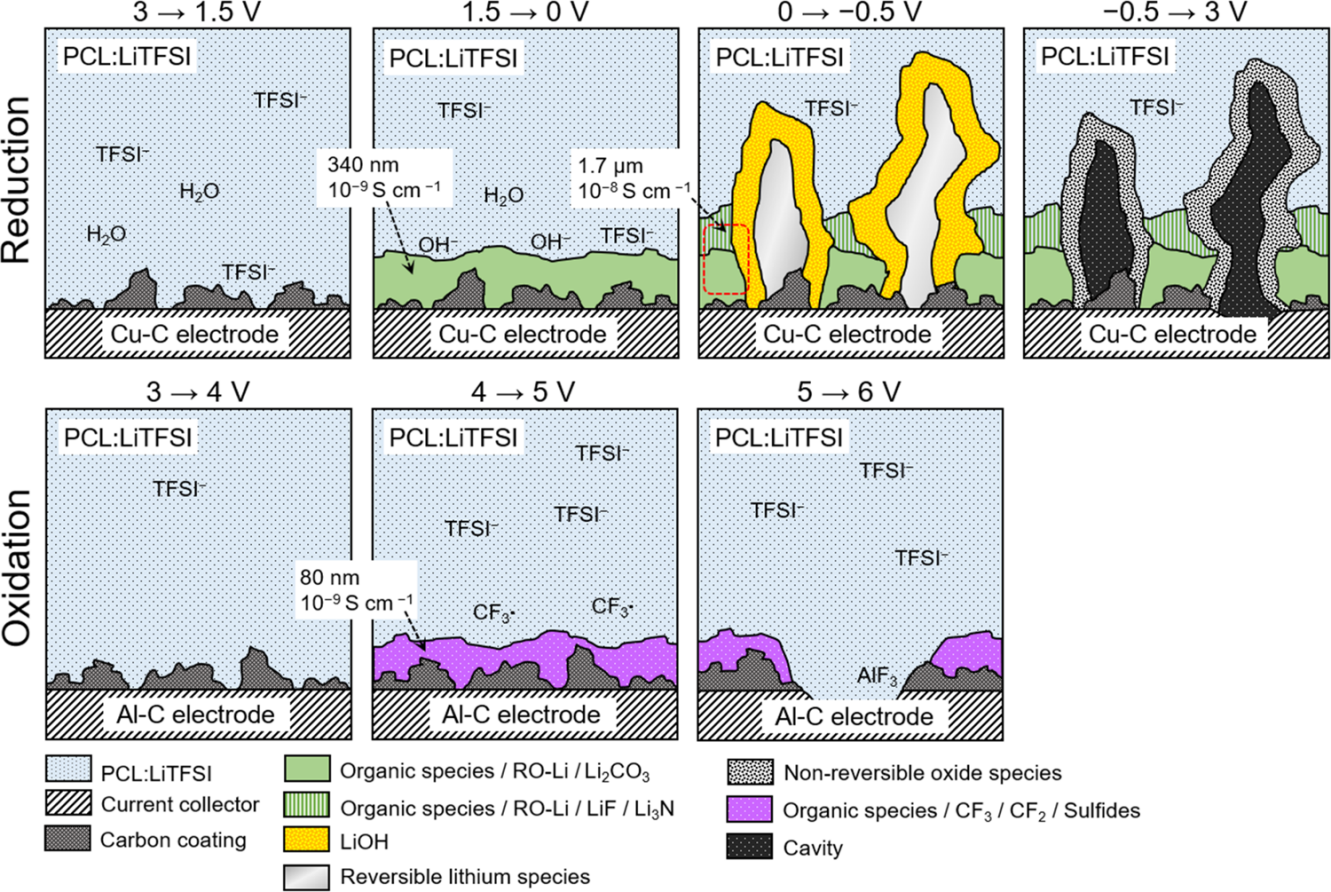
Schematic of solid polymer electrolyte–electrode interface at different potentials. Interfacial layer thickness and ionic conductivity are based on calculations that assume that the interfacial layer covers 50% of the electrode area.

The oxidation onset of PCL:LiTFSI was observed at approximately 4 V vs Li^+^/Li according to SV; however, only minor changes in the form of organic species and TFSI^–^ were observed at the interface using XPS. At 4 V, minor traces of CF_2_ fragments were observed using XPS, which indicates salt degradation. Following three consecutive oxidation cycles, a weak signal most likely belonging to polysulfides was also observed at the interface. These salt degradation species could initiate secondary side reactions with the polymer host.^30^ The absence of major interfacial species is unexpected since the impedance measurements using the three-electrode cell indicated gradual CEI formation when going to extreme potentials. This suggests that either the CEI consists of species that are indistinguishable from the polymer electrolyte and the Al–C electrode or that the subsequent decomposition of polymer electrolyte creates a region with low ionic conductivity in the vicinity of the Al–C electrode.^60,61^ This phenomenon warrants further investigation. A summary of our interpretation of the collective results in this work can be seen in Figure 9.

## Conclusions

Using a combination of complementary techniques, the electrochemical stability window of PCL with LiTFSI salt was determined to span from 1.5 to 4 V vs Li^+^/Li. Using a LiFePO_4_ reference electrode embedded in the SPE membrane, it was possible to estimate the resistance and thickness of SEI and CEI layers at potentials below and above the stability limits. The gradual increase in interfacial resistance was attributed to the accumulation of polymer- and salt-derived decomposition species, e.g., alkoxides, lithium oxides, fluorides, nitrides, and sulfides, at the SPE–electrode interface. Despite their relatively thin dimension, the interfacial layers constituted a major source of resistance in the PCL:LiTFSI system. The instability of the material at low potentials is thus a significant bottleneck for the performance of PCL-based electrolytes when implemented in battery cells unless efforts to stabilize the interface—either thermodynamically or kinetically—are undertaken.

## Supporting Information Available

Electrochemical, compositional, and morphological characterization of materials using EIS, XPS, SEM, EDS, DSC, TGA, and ^1^H NMR (PDF)
