# Supplementary material for: Dissecting the Solid Polymer Electrolyte–Electrode Interface in the Vicinity of Electrochemical Stability Limits
Source: ACS Appl Mater Interfaces. 2022 Jun 16;14(25):28716–28. doi: 10.1021/acsami.2c02118 (PMC9247984; doi:10.1021/acsami.2c02118)
Supplement: Supplementary file 1 — am2c02118_si_001.pdf [file am2c02118_si_001.pdf]

Supporting Information for

# Dissecting the solid polymer electrolyte-electrode interface in the vicinity of electrochemical stability limits

Christofer Sångeland[a], Guiomar Hernández[a], Daniel Brandell[a], Reza Younesi[a], Maria Hahlin[a,b], Jonas Mindemark[a,*]

[a]Department of Chemistry – Ångström Laboratory, Uppsala University, Box 538, SE-751 21 Uppsala, Sweden
[a]Department of Physics and Astronomy, Uppsala University, Box 516, SE-751 20 Uppsala, Sweden

*Corresponding author. E-mail: jonas.mindemark@kemi.uu.se

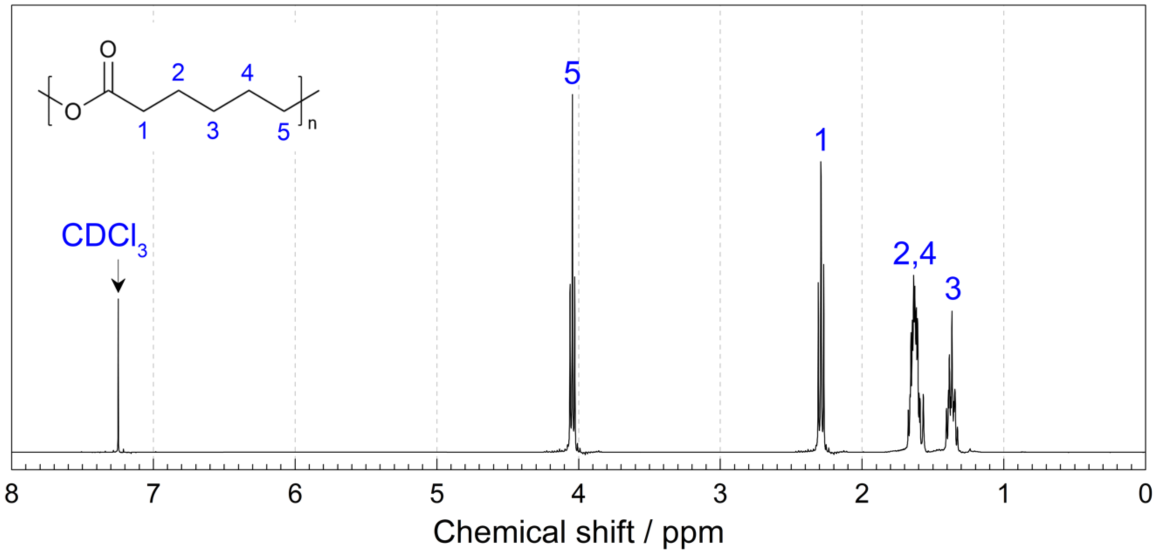


**Figure S1.** $^1$H NMR spectrum of synthesized PCL dissolved in $CDCl_3$ solvent.

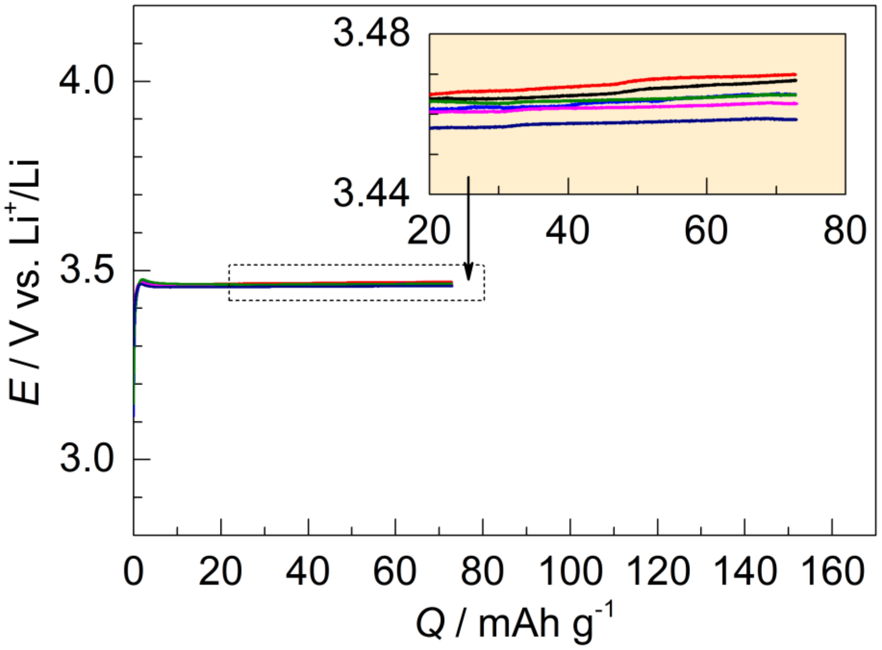


**Figure S2.** De-lithiation profiles of $LiFePO_4$ ring electrodes at charge current of 270 µA at room temperature. The cell configuration was Li | LP40/Celgard | $LiFePO_4$.

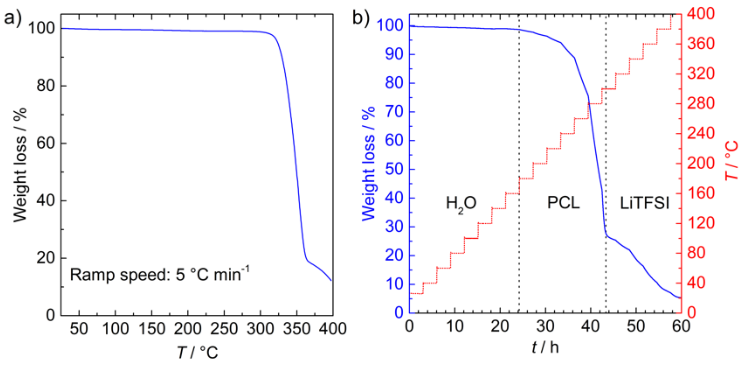


**Figure S3.** Ramping a) and stepping b) thermal gravimetric analysis of PCL:LiTFSI.

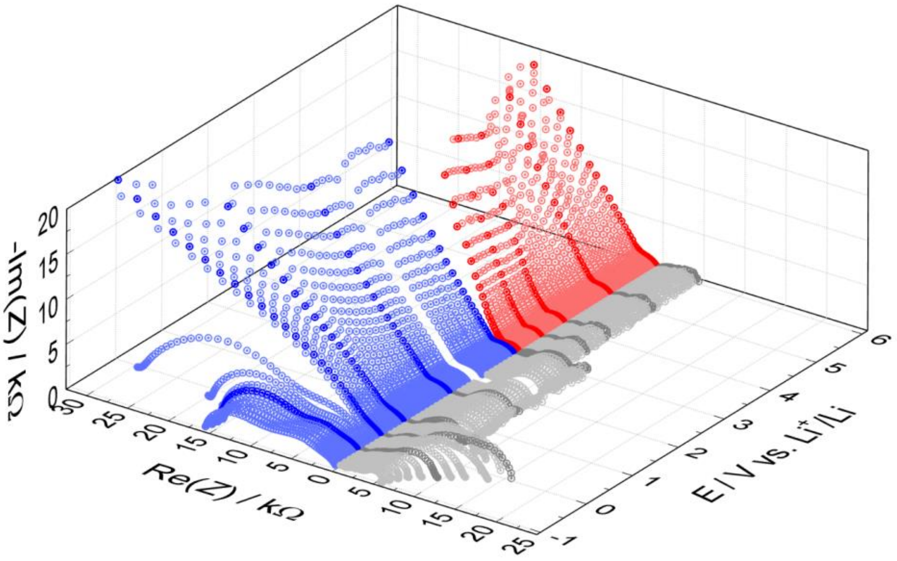


**Figure S4.** Nyquist plots of impedance response between the working electrode and the reference electrode measured 1 hour after potential steps ranging from 3.0 to −1 V and 3.0 to 6.0 V vs. $Li^+$/Li during reduction (blue) and oxidation (red), respectively. The impedance response between the counter electrode and the reference electrode (light grey) at each potential are also shown. The Nyquist plots seen in Figure 3 are highlighted in darker shades.

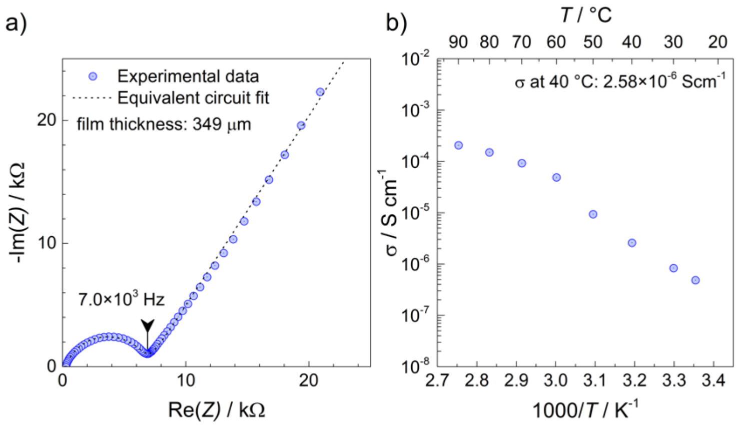


**Figure S5.** a) Nyquist plot of the impedance response of PCL:LiTFSI at 40 °C and b) total ionic conductivity of PCL:LiTFSI at temperatures ranging from 20 to 90 °C.

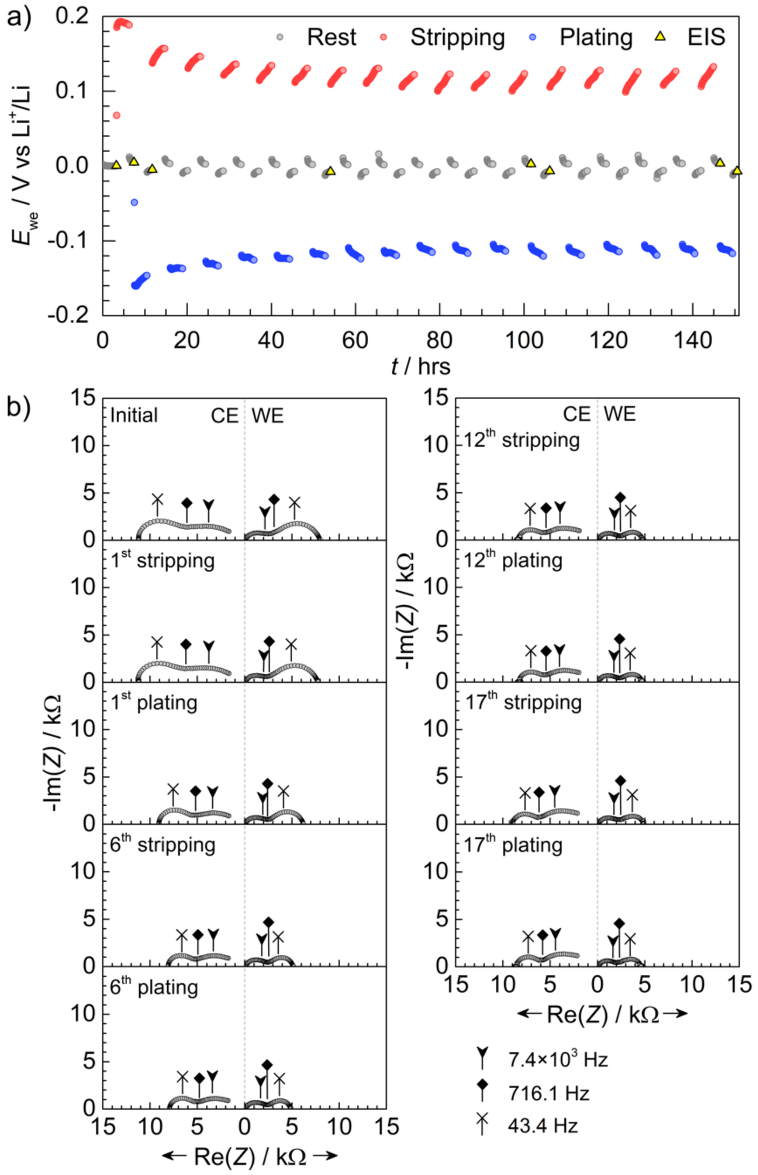


**Figure S6.** a) Lithium stripping and plating at 10 μA $cm^{-2}$ for 3 h consecutively at 40 °C and b) impedance response between the working electrode and reference electrode after each step. The impedance response between the counter electrode and the reference electrode is shown in light grey.

**Table S1.** Equivalent circuits used to fit EIS data.

| Circuit | Potential range<br>V *vs.* $Li^+$/Li | Equivalent circuit |
|---|---|---|
| Debye circuit | - | 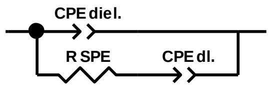 |
| A | 0–1.5<br>4.1–6 | 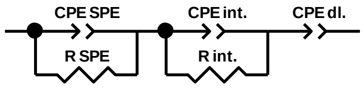 |
| B | 0–(−1) | 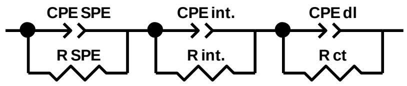 |

The equivalent circuit fits were created in ZView v.3.2b. **CPE diel.**: dielectric capacitance of the polymer electrolyte; **R SPE**: bulk ionic resistance of the polymer electrolyte; **CPE dl.**: double layer capacitance at the electrode–polymer interface; **CPE SPE**: capacitance of the polymer electrolyte; **CPE int.**: capacitance of the interphase layer; **R int.**, resistance of the interphase layer; **R ct.**: charge transfer resistance.

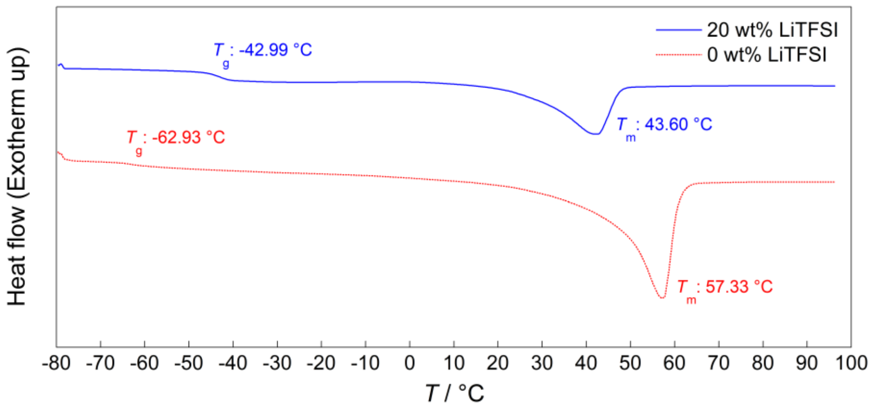


**Figure S7.** DSC thermal scans for high molecular weight poly(caprolactone) with and without 20 wt% LiTFSI.

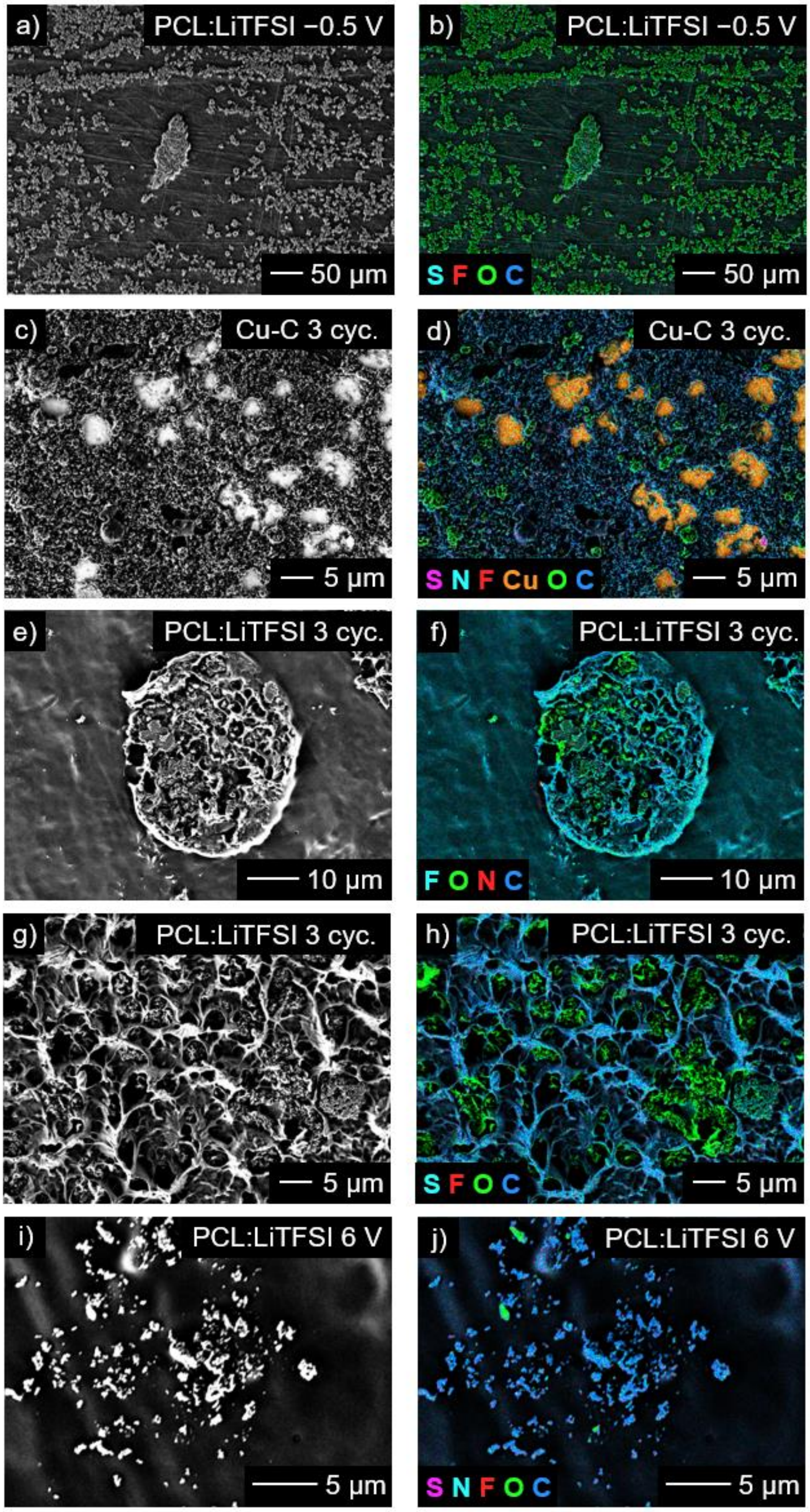


**Figure S8.** SEM and EDS micrographs of a,b) PCL:LiTFSI surface after reduction to −0.5 V *vs.* $Li^+$/Li; c,d) Cu-C surface following 3 cycles from OCV to −0.5 V; e–h) PCL:LiTFSI surface after 3 cycles from OCV to −0.5 V; and i,j) PCL:LiTFSI surface following oxidation to 6 V.

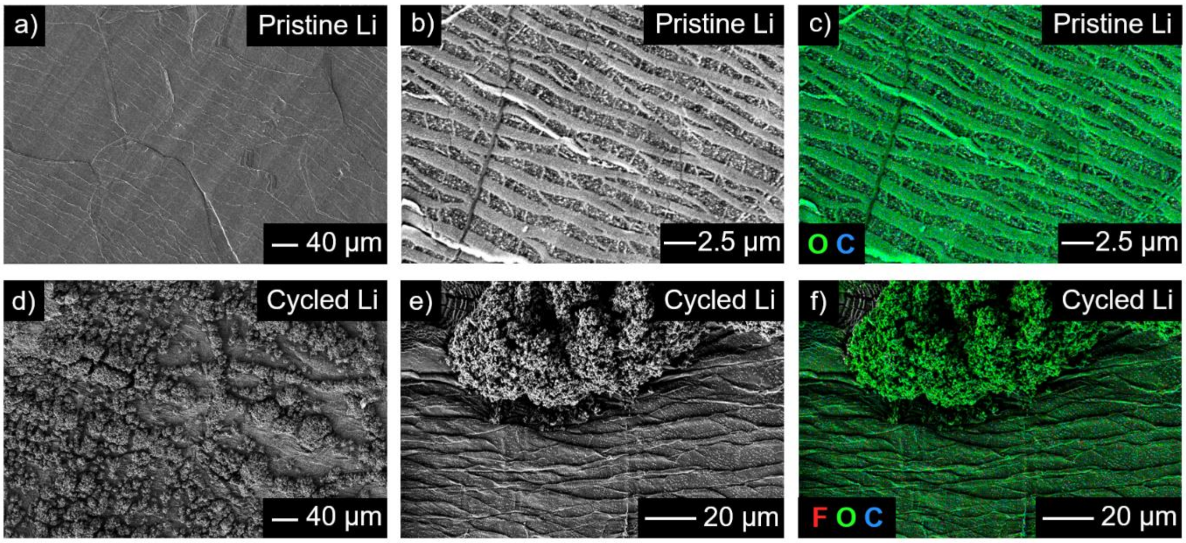


**Figure S9.** SEM and EDS micrographs of a–c) pristine lithium foil and d–f) lithium foil after 17 stripping and plating cycles.

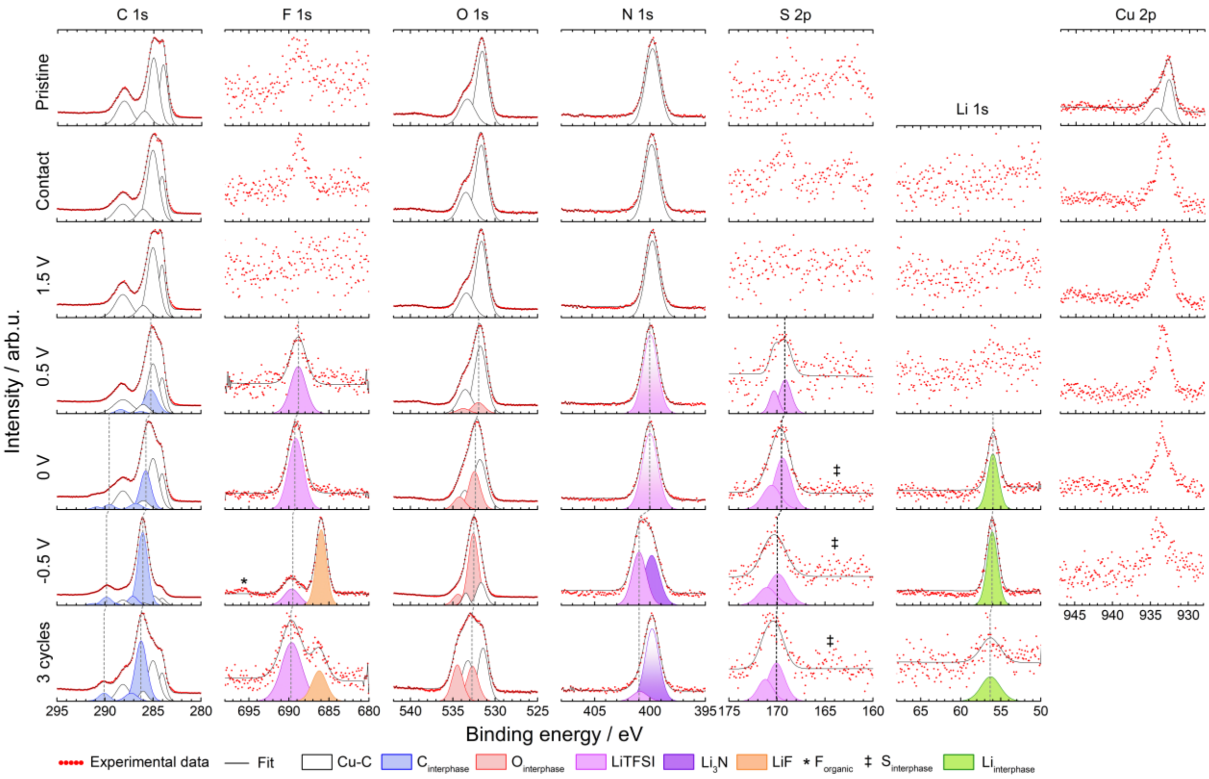


**Figure S10.** XPS spectra from the Cu-C surface at different stages: prior to assembly (pristine), following contact (contact), at 1.5, 0.5, 0, −0.5 V *vs.* $Li^+/Li$ and after 3 cycles from OCV to −0.5 V. Spectra were normalized according to the highest intensity count in each spectrum.

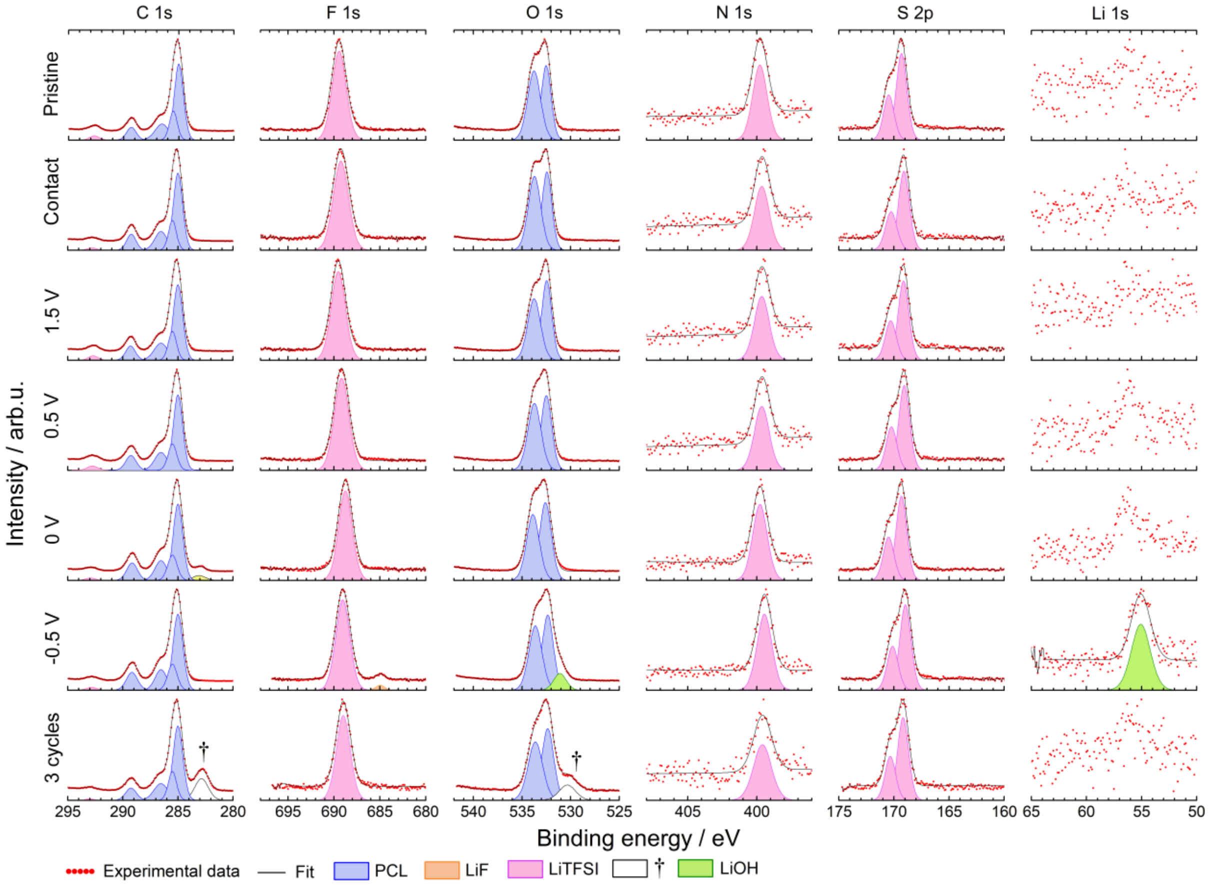


**Figure S11.** XPS spectra from the PCL:LiTFSI surface facing Cu-C at different stages: prior to assembly (pristine), following contact (contact), at 1.5, 0.5, 0, −0.5 V vs. $Li^{+}/Li$ and after 3 cycles from OCV to −0.5 V . Spectra were normalized according to the highest intensity count in each spectrum.

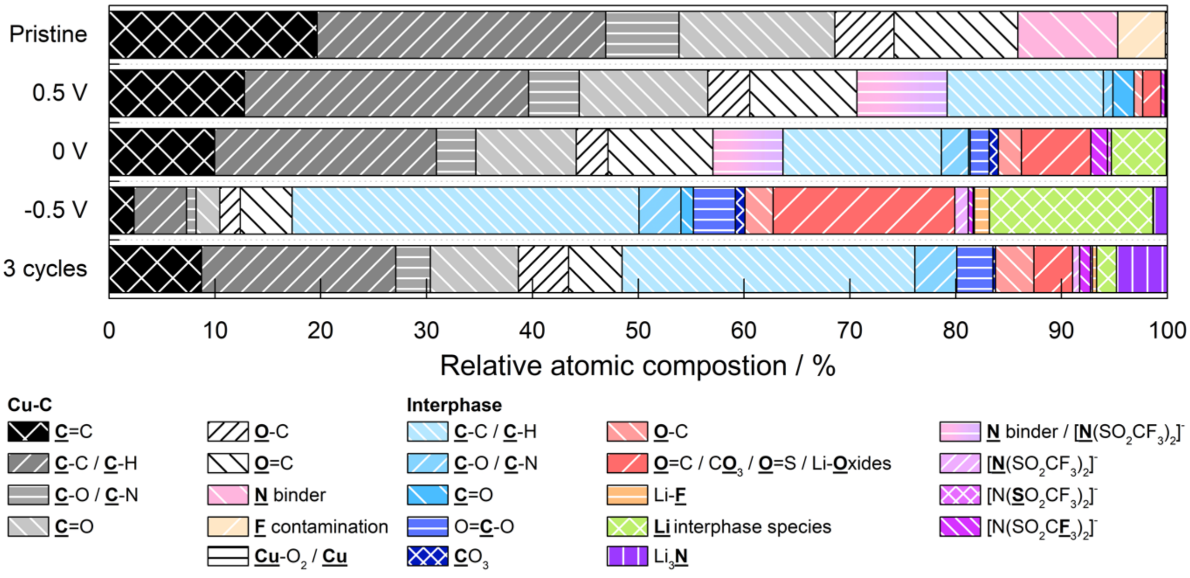


**Figure S12.** Relative atomic composition of the Cu-C surface prior to assembly (pristine), at 0.5, 0, −0.5 V *vs.* $Li^{+}/Li$ and after 3 cycles from OCV to −0.5 V. Given the surface sensitivity of XPS and surface inhomogeneity of the samples, as indicated by SEM micrographs, it should be emphasized that these quantities give a somewhat distorted representation of the interface composition.

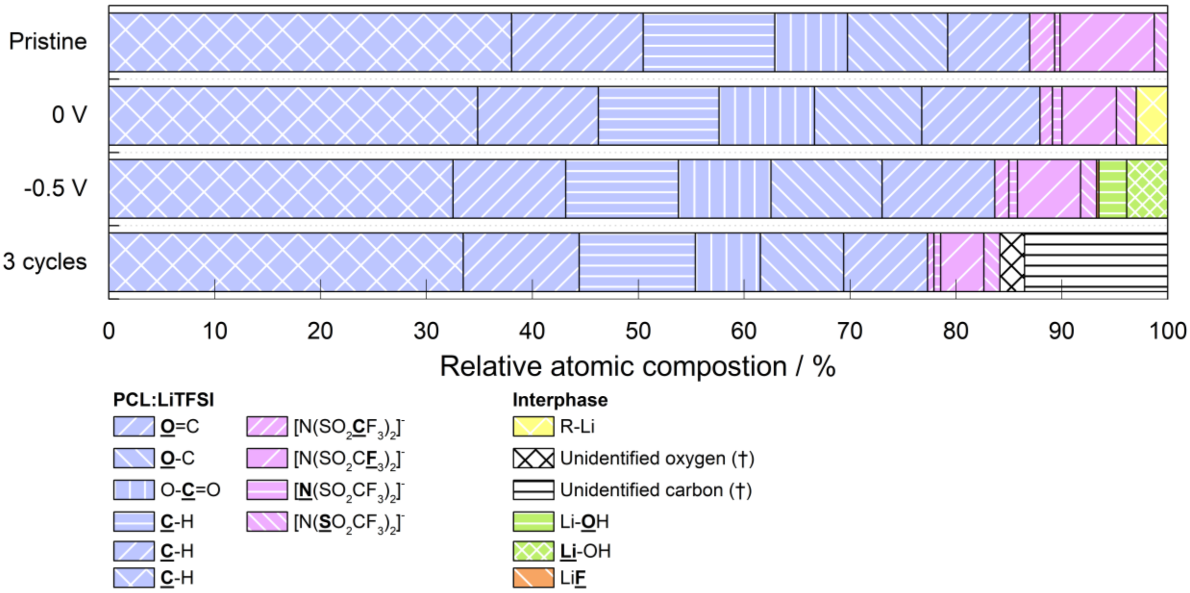


**Figure S13.** Relative atomic composition of the Cu-C surface prior to assembly (pristine), at 0.5, 0, −0.5 V *vs.* $Li^+/Li$ and after 3 cycles from OCV to −0.5 V. Given the surface sensitivity of XPS and surface inhomogeneity of the samples, as indicated by SEM micrographs, it should be emphasized that these quantities give a somewhat distorted representation of the interface composition.

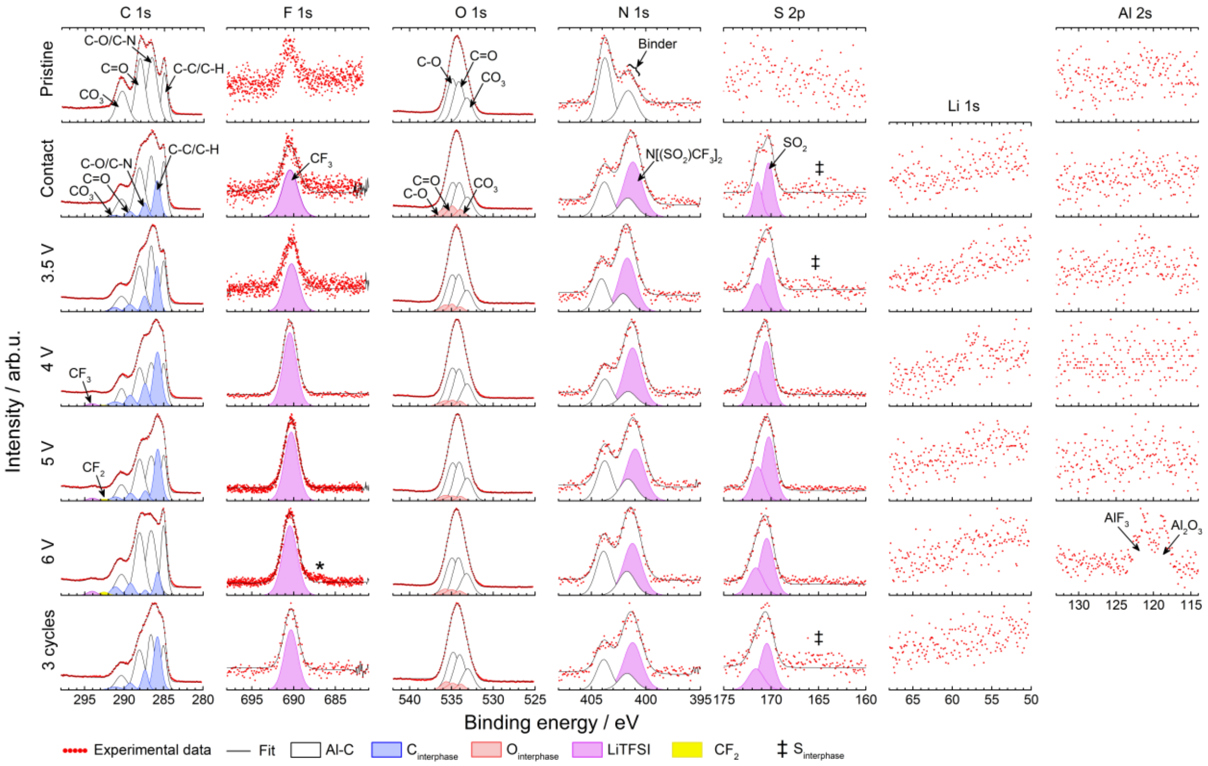


**Figure S14.** XPS spectra from the Al-C surface at different stages: prior to assembly (pristine), following contact (contact), at 3.5, 4, 5, 6 V *vs.* $Li^+/Li$ and after 3 cycles from OCV to 5 V. Spectra were normalized according to the highest intensity count in each spectrum.

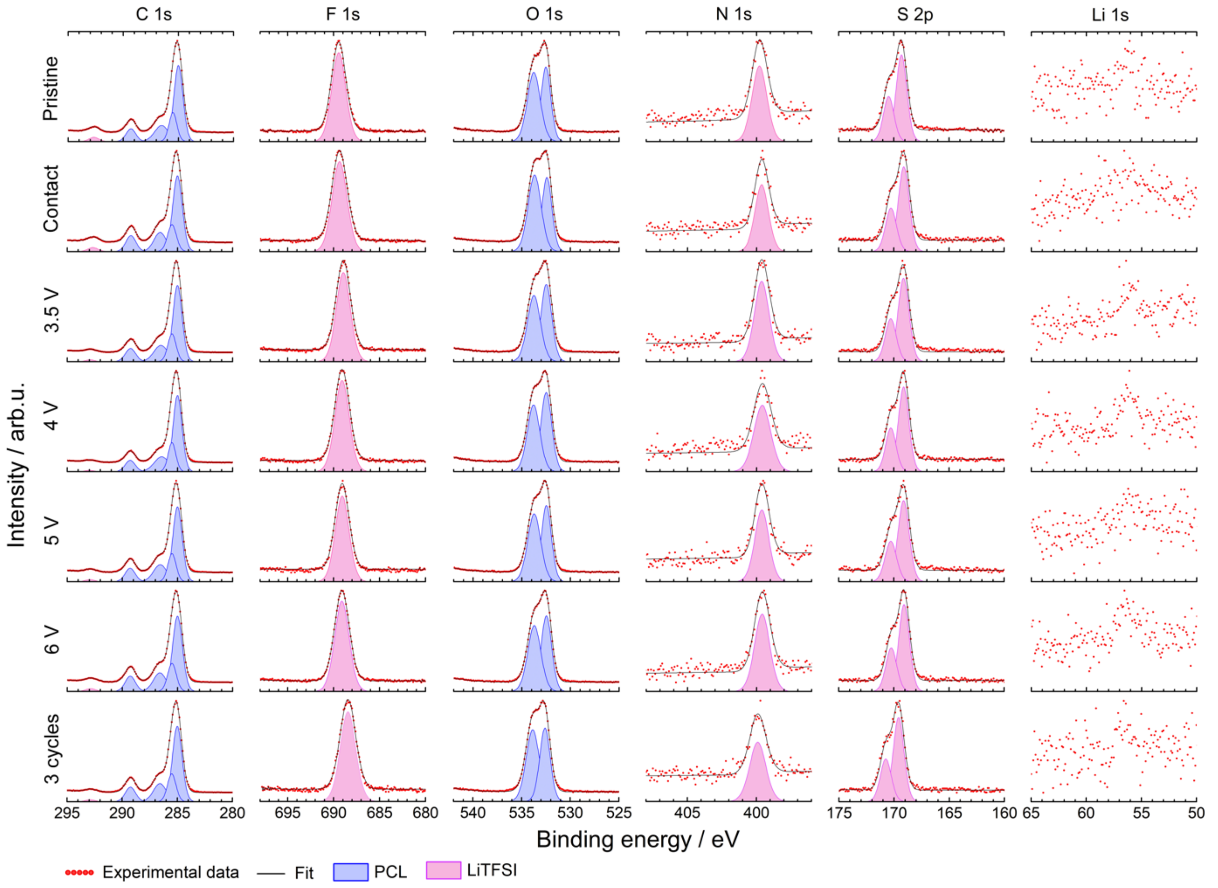


**Figure S15.** XPS spectra from PCL:LiTFSI facing Al-C at different stages: prior to assembly (pristine), following contact (contact), at 3.5, 4, 5, 6 V *vs.* $Li^+$/Li and after 3 cycles from OCV to 5 V. Spectra were normalized according to the highest intensity count in each spectrum.
